# Artificial Intelligence Applied to Improve Scientific Reviews: The Antibacterial Activity of *Cistus* Plants as Proof of Concept

**DOI:** 10.3390/antibiotics12020327

**Published:** 2023-02-04

**Authors:** Francisco Javier Álvarez-Martínez, Fernando Borrás-Rocher, Vicente Micol, Enrique Barrajón-Catalán

**Affiliations:** 1 Instituto de Investigación, Desarrollo e Innovación en Biotecnología Sanitaria de Elche (IDiBE), Universidad Miguel Hernández (UMH), 03202 Elche, Spain; 2 Statistics and Operative Research Department, UMH, Avda, Universidad s/n, 03202 Elche, Spain; 3 CIBER, Fisiopatología de la Obesidad y la Nutrición, CIBERobn, Instituto de Salud Carlos III (CB12/03/30038), 28029 Madrid, Spain; 4 Department of Pharmacy, Elche University Hospital-FISABIO, 03203 Elche, Spain

**Keywords:** *Cistus*, antibacterial, artificial intelligence, natural language processing, NLP, clustering, patent

## Abstract

Reviews have traditionally been based on extensive searches of the available bibliography on the topic of interest. However, this approach is frequently influenced by the authors’ background, leading to possible selection bias. Artificial intelligence applied to natural language processing (NLP) is a powerful tool that can be used for systematic reviews by speeding up the process and providing more objective results, but its use in scientific literature reviews is still scarce. This manuscript addresses this challenge by developing a reproducible tool that can be used to develop objective reviews on almost every topic. This tool has been used to review the antibacterial activity of *Cistus* genus plant extracts as proof of concept, providing a comprehensive and objective state of the art on this topic based on the analysis of 1601 research manuscripts and 136 patents. Data were processed using a publicly available Jupyter Notebook in Google Collaboratory here. NLP, when applied to the study of antibacterial activity of *Cistus* plants, is able to recover the main scientific manuscripts and patents related to the topic, avoiding any biases. The NLP-assisted literature review reveals that *C. creticus* and *C. monspeliensis* are the first and second most studied *Cistus* species respectively. Leaves and fruits are the most commonly used plant parts and methanol, followed by butanol and water, the most widely used solvents to prepare plant extracts. Furthermore, *Staphylococcus. aureus* followed by *Bacillus. cereus* are the most studied bacterial species, which are also the most susceptible bacteria in all studied assays. This new tool aims to change the actual paradigm of the review of scientific literature to make the process more efficient, reliable, and reproducible, according to Open Science standards.

## 1. Artificial Intelligence Applied to Language Processing in *Cistus* Research

Scientists and academics are often faced with literature reviews on topics that span hundreds or even thousands of articles. The time and effort required to search, refine, collect, read, and synthesize all the information relevant to the task can often be overwhelming. In addition, this situation is frequently influenced by authors’ background, experience, and knowledge, leading to selection bias that can lead to different versions of the state-of-the-art depending on the authors. For this reason, this work addresses the issue more efficiently and objectively using technological tools that are easily accessible today and based on artificial intelligence applied to natural language processing (NLP). The benefits of the different NLP based tools in the reviewing process are numerous and clear. In this sense, there are numerous studies addressing the different steps of the process [1,2] as well as many online available tools that can help researchers to carry on this difficult task. But despite the advances of the algorisms and tools, there is a lack of studies using NLP to carry on reviews on specific topics as a specific biological activity as our study aims to do. On the other hand, most of these new tools are quite opaque and do not allow the user to interact with the algorithm and guide the real review process, resulting non attractive for most of the users who are used to work with more classical tools. According with these premises novelty, objectivity, and usability, this study presents a simple online tool that is fully editable by users and adaptable to any topic, providing not only a selection and clusterization of the main relevant published studies, but also complementary information about the historical trend of the topic, the patents, citations, and open access policy status. These additional resources allow the user to compare and analyze the state-of-the-art in a more organized and objective way, leading to a more reliable review process.

To obtain and process the existing bibliography related to the genus *Cistus*, on 26 May 2022, a search was carried out in the Scopus database using the term “*Cistus*” within “article title, abstract, keywords” default search and without applying any filter or restriction, generating a total of 1601 research manuscripts. Due to the vast amount of information obtained and the enormous time it would take to analyze and classify each paper, it was decided to use artificial intelligence applied to NLP to automatically analyze and classify the different papers based on the content of their titles and abstracts.

Although there are other recent reviews on the genus *Cistus* [3], the present work selects and categorizes the information in a completely objective and comprehensive way, including information from both research articles and patents. This is a completely new approach, for *Cistus* plants research, with a view to contributing to the collective effort of the scientific community to access the generated knowledge simply and effectively. Furthermore, the online tools that have been developed here are fully open access to be used by the scientific community and completely adaptable to any research topic.

## 2. Plants of the Genus *Cistus*

Plants belonging to the genus *Cistus* L. (from the Greek word *kistos*), also known as rockrose or “jara” in Spanish, are herbaceous perennial dicotyledonous plants predominant in the Mediterranean region. This genus is made up of various pioneer species, characterized by their ability to proliferate in areas exposed to drought, with stony and infertile soils [4]. These are very resistant pyrophilic plants that are able to regenerate quickly after forest fires due to the seeds’ increased germination capacity after exposure to high temperatures [5].

The genus *Cistus* is one of eight genera that comprise the family *Cistaceae* L. This genus is relatively small and biologically diverse due to high levels of polymorphism and hybridization between related species due to cross-pollination [6]. Studies to date recognize between 16 and 28 different species belonging to the genus *Cistus*, highlighting the intraspecific conflicts underlying their classification [7]; furthermore, there are 31 *Cistus* species currently listed in the Euro+Med PlantBase [8]. According to their ethnobotanical use, previous scientific studies, and ecological relevance, the eight most relevant identified species that make up the genus are *Cistus albidus* L., *Cistus creticus* L., *Cistus incanus* L., *Cistus crispus* L., *Cistus ladanifer* L., *Cistus monspeliensis* L*., Cistus parviflorus* Lam., and *Cistus salviifolius* L. [9].

Regarding their plant physiology, the branches of these species range between 50 and 100 cm in length, with corrugated and opposite leaves between 2 and 8 cm in length. Their visible flowers are hermaphrodite, actinomorphic, and hypogynous, with three of their five sepals opposite the petals [10]. Petal color ranges from white to dark purple/pink depending on the subgenus, with a conspicuous dark red spot at the base of each petal present in some species. The sweet-smelling fragrance of some species makes them highly prized in the perfume industry [11].

### 2.1. Research on the Genus Cistus in Numbers

#### 2.1.1. Article Clustering

**Figure 1:**
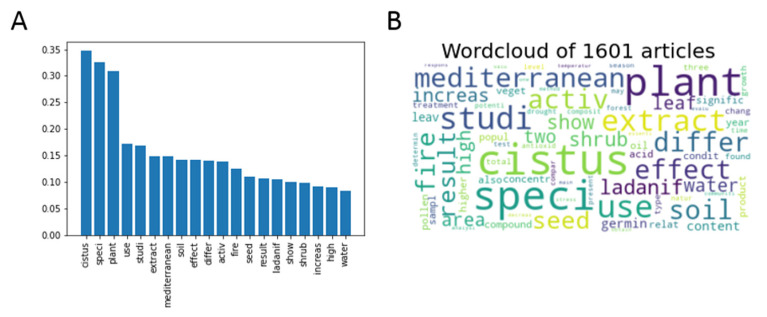
(**A**) Bar graph with the relative frequencies of the 20 most abundant words in the analyzed bibliography. (**B**) Word cloud as a visual representation of the frequency of the 75 most common words. A larger font size indicates a higher relative frequency of occurrence.

The information on the language contained in the 1601 articles extracted from Scopus related to *Cistus* was processed and analyzed using the Jupyter notebook on Google Colab mentioned in the “Methodology” section, which includes a link. All the words were processed until their root was obtained to avoid deviations due to the different forms of the same word. Words with no intrinsic meaning (prepositions, connectors, articles, etc.,) were removed to gain better insight into the essence and meaning of the text. After this first analysis, the most frequent words contained in the articles were obtained (Figure 1), and some were retrieved as the root of a word’s family, such as “anali” (for the family containing analysis, analytical, analyzed, etc.,). As expected, *Cistus*, followed by the two very general terms “speci” and “plant”, were the three most retrieved, twice as often as any of the other terms.

A more detailed analysis of the obtained results revealed other frequent general terms, such as “use” “studi”, “mediterranean” and “extract”, and no specific terms. However, other more specific terms, such as “fire”, “ladanif”, “soil”, and “seed” that may represent specific research areas deserve to be deepened. Accordingly, it was decided to cluster the articles based on their thematic similarities using language equivalencies.

**Figure 2:**
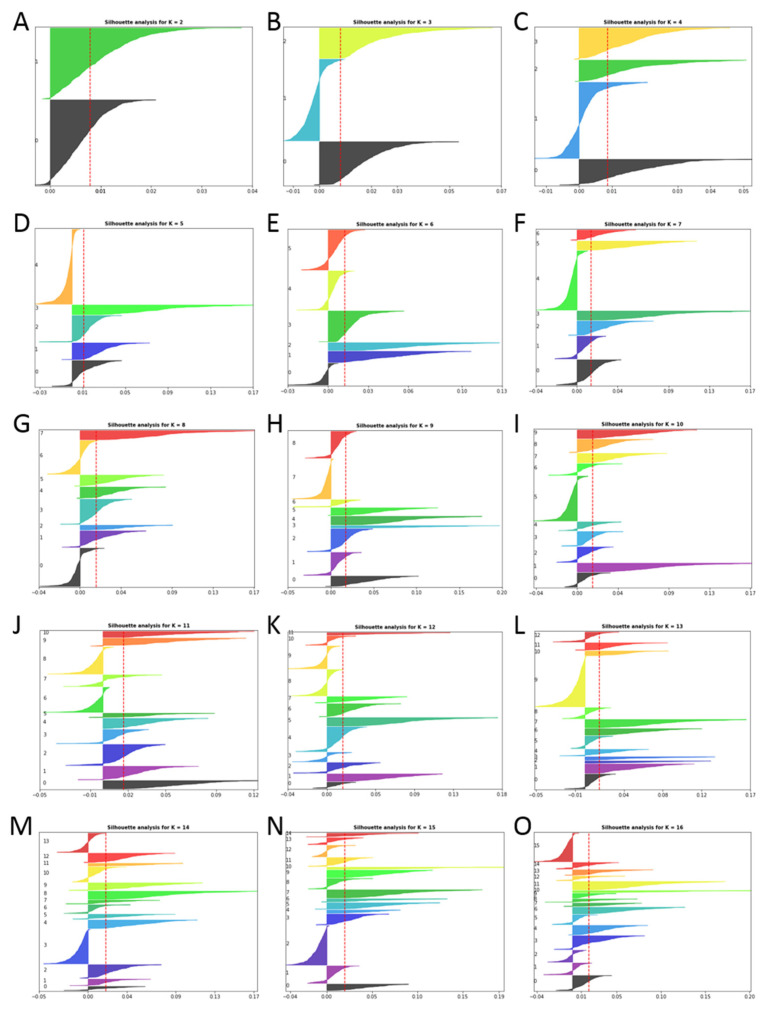
Silhouette plots for the different cluster numbers tested for the *Cistus* articles dataset up to 16. (**A**–**O**) show the results for grouping the articles in K groups (K value for each figure is shown in the upper part of each plot).

To cluster the articles, the silhouette score method was used, in which silhouette values are calculated based on the similarity of the article’s language to a certain thematic cluster (cohesion) compared to the rest of the clusters (separation). This method is useful to determine the optimal number of clusters to group the information. Figure 2 shows the silhouette graphs for the separation of 2 into 16 different clusters.

**Figure 3:**
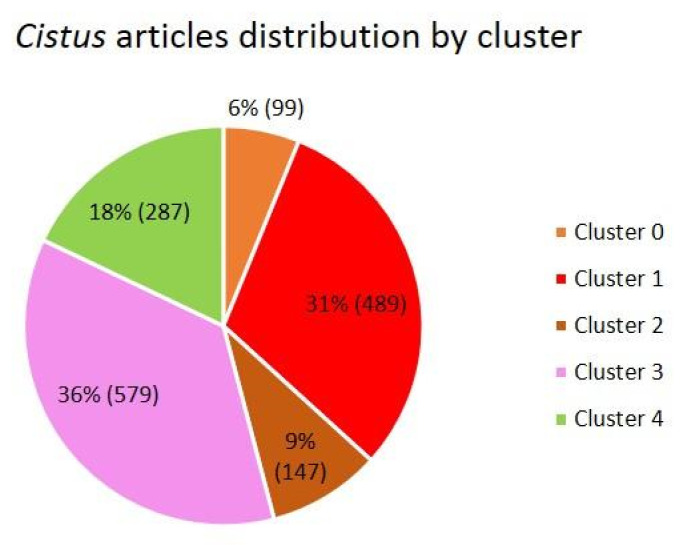
Pie chart with the distribution of articles in each of the clusters presented as a percentage and as a total number in parentheses.

For the purpose of this work, it was estimated that the optimal number of clusters in which to divide the dataset was 5 (Figure 2D), and they were numbered from 0 to 4. The number of clusters was selected based on the silhouette graphs, ensuring that the silhouettes of each cluster exceeded the red dotted line (average silhouette score) and that there was the greatest parity between the thickness of each silhouette (see further details in Methodology section). Taking these factors into account, 5 was estimated to be the optimal number of clusters. For this number of clusters, a homogeneous and coherent thematic separation was observed. Figure 3 shows the quantitative distribution of the articles in the different clusters.

**Figure 4:**
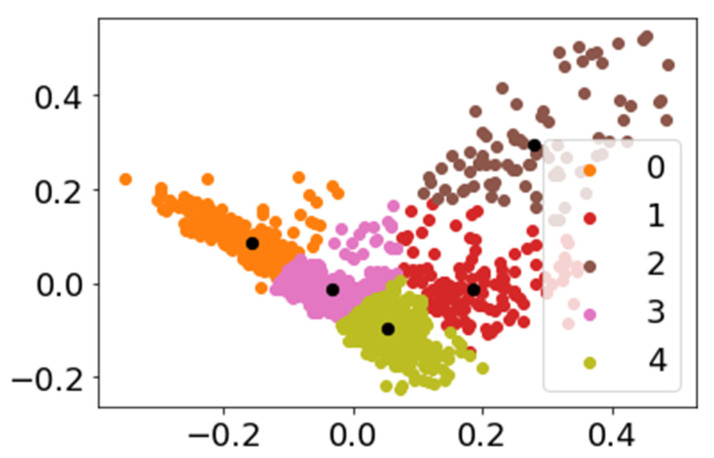
Scatter plot from PCA of the 1601 articles collected in this work. The different colors represent the grouping of items in each of the 5 clusters. The black dots represent the centroids of each cluster.

To visualize the complexity of the dataset and its clustering, a principal component analysis (PCA) was used to obtain a 2D scatter plot showing the relationships among the different clusters (Figure 4).

As seen in Figure 4, Clusters 0, 3, and 4 are close to each other, in addition to presenting little dispersion in the points, which are close to the centroids (black dots). This means that these clusters contain very cohesive and similar information that is also somehow related regarding the language and terms used. Cluster 1 is also related to the previous clusters, especially to Cluster 4, but a greater dispersion can be observed, indicating that different terms and information are included. This correlation makes sense since Cluster 4 addresses the issue of growth capacity in soils with the presence of metals, and this is related to the antioxidant activity of the compounds present in *Cistus*, a subject included in Cluster 1. On the other hand, Cluster 2 brings together a greater variety of articles and presents the greatest dispersion and the greatest distance with respect to the remaining clusters.

**Figure 5:**
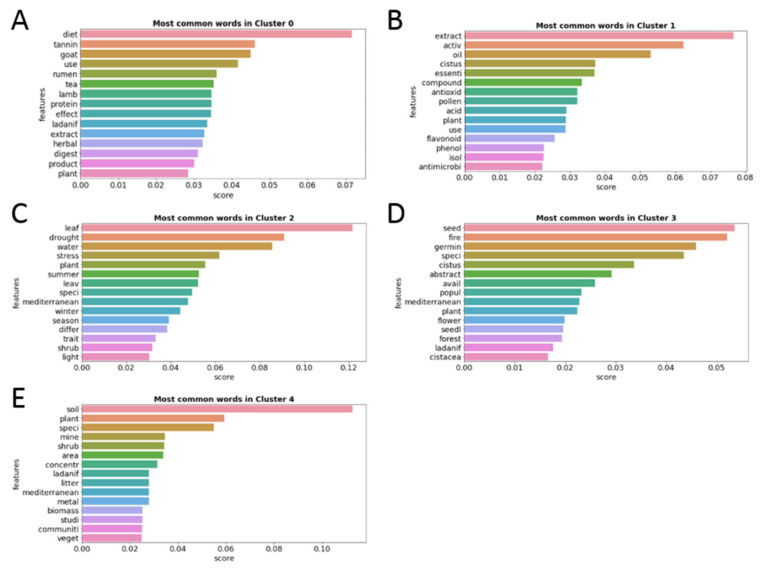
Fifteen words with the highest relative frequency of appearance in each of the five article clusters. Each cluster is identified on the top of each figure (**A**–**E**).

The most common words of each of the 5 clusters were analyzed to determine the research topic of the articles included in each of them. Figure 5 shows the 15 words with the highest relative frequency of appearance.

The results of the analysis of the language of the clusters provide an idea of the main research topic covered by each of them. Cluster 0, the smallest cluster with only 6% of the total articles (99 articles), brings together articles that deal mainly with the effect of tannins present in plants of the *Cistus* genus, especially *C. ladanifer*, that certain ruminants, such as goats, consume in their diet, since there are indications that relate the levels of these phenols with better health and quality of these animals’ meat and milk [12,13,14,15]. Cluster 1, the second-largest cluster with 31% of the total articles (489 articles), groups articles that address the bioactive and medicinal properties of the extracts and essential oils obtained from *Cistus*, such as its antioxidant and antibacterial capacity [6,16,17]. Cluster 2, with 9% of the total articles (147 articles), includes articles related to plant physiology and growing conditions [18,19,20,21]. Cluster 3, the largest cluster with a total of 36% of the total articles (579 articles), addresses the growing conditions, ecology, and landscape regenerative capacity of plants of the *Cistus* genus, especially after fires [22,23,24,25]. Finally, Cluster 4, with the remaining 18% of articles (287 articles), delves into ecosystem enhancement and the relationship between plants of the *Cistus* genus and the different types of soil, with a special emphasis on how these plants are able to grow and regenerate metal-contaminated soils [26,27,28,29].

Once the articles are distributed in the thematic clusters, it is possible to obtain the articles of the most interesting cluster(s) for the researcher and then proceed to a more detailed subclustering, as described in Section 2.1.6. Alternative analyses can also be developed, as described in the following sections (Section 2.1.2, Section 2.1.3, Section 2.1.4 and Section 2.1.5).

#### 2.1.2. Trends in the Last 20 Years

**Figure 6:**
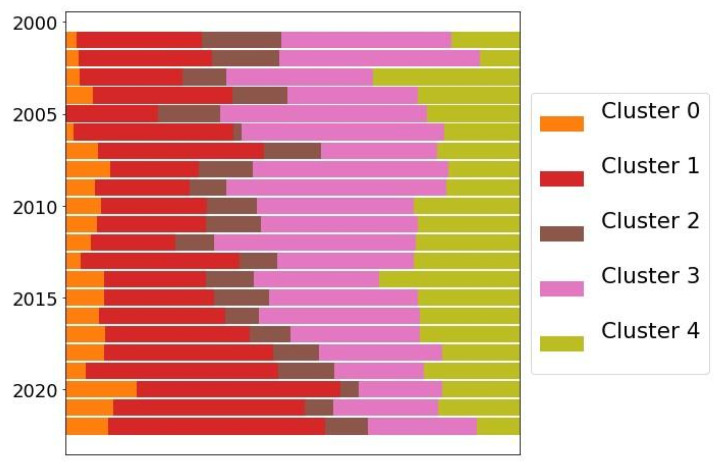
Evolution graph of the size of the different clusters of articles on *Cistus* from 2001 to 2021. The first months of 2022 are also included.

The clustering of the articles also serves to track trends in research during certain periods of time. For example, it is possible to visualize which clusters have predominated in *Cistus* research during the last 20 years (Figure 6), also observing their evolution over time. This analysis is useful for determining the volume of research work in a particular field or application and the interest it generates. In the present work, it is useful to follow the trend of Cluster 1 regarding the bioactivity of the *Cistus* genus, which includes its antibacterial capacity.

In Figure 6, Clusters 0 and 1 show a growing trend, which denotes the upward trend of scientific interest, especially in the case of Cluster 1, which has now become the majority. Clusters 2 and 4 maintained a certain stability during the observed period. Cluster 3 presents a gradual decrease, from being the most studied in 2001 to the second-most studied in 2021, far from Cluster 1 and closely followed by Cluster 4.

#### 2.1.3. Open Access Cistus Research Analysis

Open access journals have significantly changed the way research is published, but what about the science itself? Is the topic of interest influenced by the open access, or are topics addressed in the same way regardless of the publishing policy? The tool that has been developed here allows us to observe differences between groups of articles based on characteristics such as their availability in Open Access. In this way, the 1601 articles compiled in this review were divided into two groups: Group A—articles published in Open Access (424 articles) and Group B—articles published under subscription systems or similar systems other than Open Access (1177 articles).

**Figure 7:**
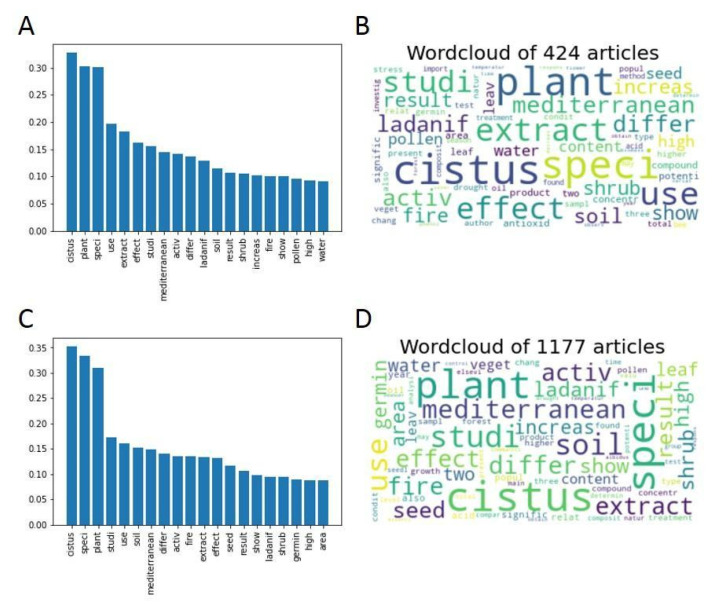
Top 20 most frequent words (**A**) and word clouds (**B**) of the articles present in Group A (published in Open Access). Top 20 most frequent words (**C**) and word clouds (**D**) of the articles present in Group B (not published in Open Access).

Figure 7 compares the words with the highest frequency in both groups in the forms of graphs and word clouds.

Comparing the word frequency graphs reveals that among the 20 most frequent words in Groups A and B, 17 are common to both, in addition to presenting similar relative frequencies. The three different words in Group A are “increase” (position 15 in the frequency graph), “pollen” (position 18), and “water” (position 20), while the three different words in Group B are “seed” (position 13), “germin” (position 18) and “area” (position 20). These words appear with low relative frequencies within the ranking. The only thematic difference between the two groups seems to be that Group B of non-Open Access articles contains more information on seeds and germination than Group A of Open Access articles. The similarity between the groups can also be observed qualitatively in the word clouds. It can be concluded that there are no major thematic differences between the articles published in Open Access and those of other non-Open Access publication systems.

To corroborate the similarity between the groups, the articles of Groups A and B were subclustered to observe in detail the thematic units that they cover. The silhouette score method was used to determine the optimal number of clusters. It was determined that the optimal division for the articles was five clusters for both Group A (Supplementary Materials Figure S1D) and Group B (Supplementary Figure S2D).

**Figure 8:**
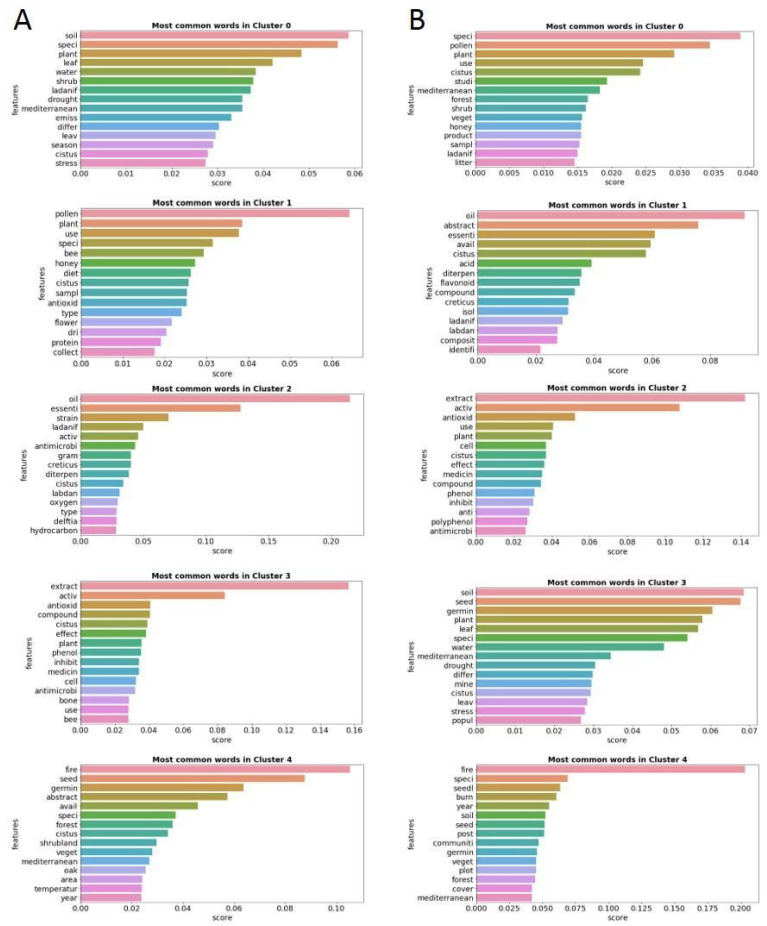
Column (**A**): Frequency graphs with the 15 most common words of each of the five clusters of Group A made up of Open Access articles. Column (**B**): Frequency graphs with the 15 most common words of each of the five clusters of Group B made up of non-Open Access articles.

Figure 8 shows the most frequent words of the five clusters of Group A in the lefthand column and the most frequent words of the five clusters of Group B in the righthand column.

Observing the themes of the subclusters of Groups A and B, the great thematic similarity between the groups can be corroborated. The most frequent words in the clusters of Group A are “soil” in Cluster 0, “pollen” in Cluster 1, “oil” in Cluster 2, “extract” in Cluster 3 and “fire” in Cluster 4. The clusters of Group B have the most frequent main words “speci” in Cluster 0, closely followed by “pollen” and “Oil” in Cluster 1, “extract” in Cluster 2, “soil” in Cluster 3 and “fire” in Cluster 4. The thematic identity of the clusters is practically identical in both groups, with a single difference for Cluster 0, corroborating the results of the previous analysis.

In conclusion, there is no thematic difference between the research articles on the genus *Cistus* published in Open Access mode with respect to other publication options.

#### 2.1.4. Most Cited Cistus Research Analysis

Another interesting parameter that can be analyzed is comparing the topics covered in the most cited articles in *Cistus* research to those in less cited articles. To carry out this analysis, the 1601 articles were divided into two groups: Group A—20% of the most cited articles (319 articles) and Group B—80% of the remaining articles (1282 articles). In the group of most cited articles, there are articles with 38 to 620 citations, with a mean of 77.12 citations and a median of 60 citations. In the second group, there are articles with 0 to 38 citations, with a mean of 11.47 citations and a median of 9 citations.

**Figure 9:**
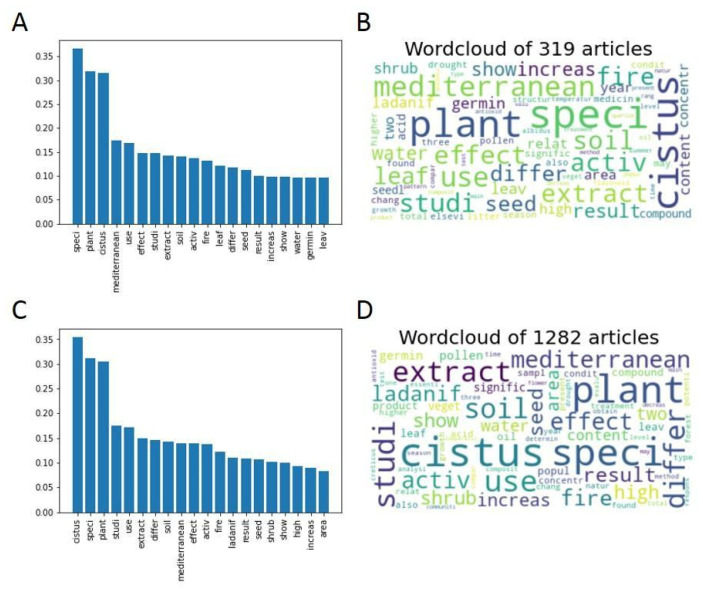
Top 20 most frequent words (**A**) and word clouds (**B**) of the articles present in Group A (top 20% most cited articles). Top 20 most frequent words (**C**) and word clouds (**D**) of the articles present in Group B (the rest of the articles).

Figure 9 compares the words with the highest frequency in both groups in the forms of graphs and word clouds.

Comparing the word frequency graphs shows that among the 20 most frequent words in Groups A and B, 16 are common to both, in addition to presenting similar relative frequencies. The four different words in Group A are “leaf” (position 12 in the frequency graph), “water” (position 18), “germin” (position 19) and “leav” (position 20), while the four different words in Group B are “ladanif” (position 13), “shrub” (position 16), “high” (position 18) and “area” (position 20). The words that differ between the groups appear with low frequency, between positions 16 and 20. However, both the word “leaf” in position 12 of Group A and the word “ladanif” in position 13 of Group B are absent in the other group. This observation indicates that in Group A of the most cited articles, more articles are written about leaves, while in the group of less cited articles, more articles related to the species *C. ladanifer* are found.

Groups A and B were clustered to observe in detail the thematic units that they cover. The silhouette score method was used to determine the optimal number of clusters. It was determined that six was the optimal number of clusters for the articles of Group A (Supplementary Figure S3E) and Group B (Supplementary Figure S4E).

**Figure 10:**
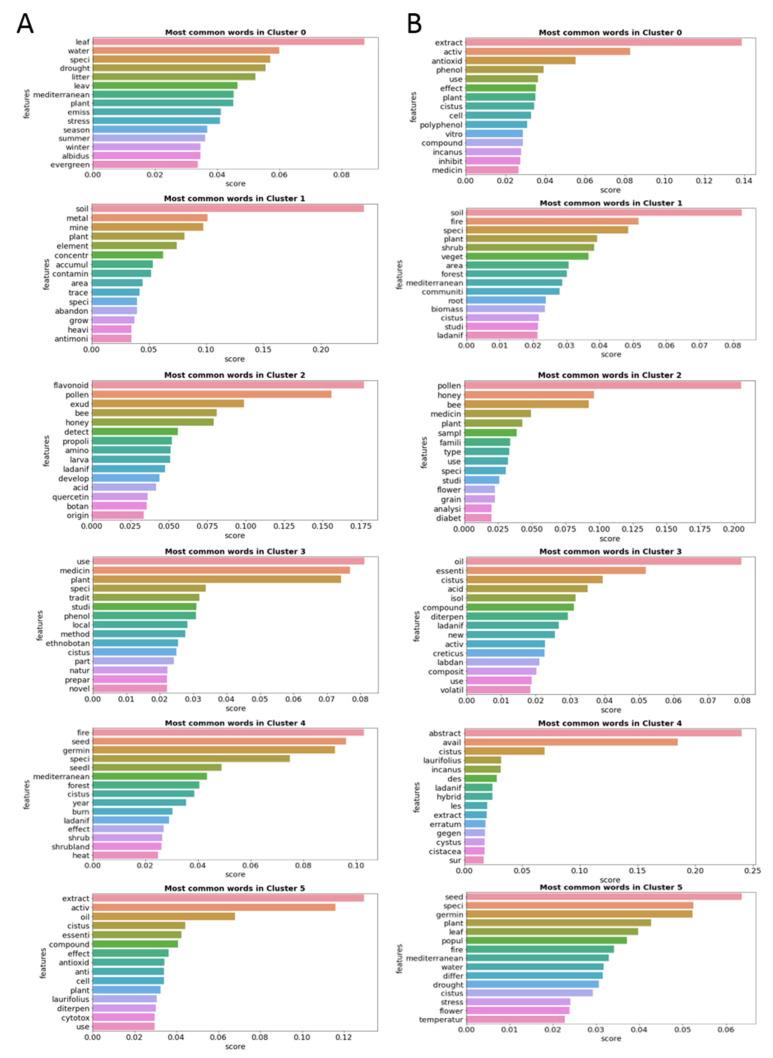
Column (**A**): Frequency graphs with the 15 most common words of each of the six clusters of Group A made up of the top 20% most cited articles. Column (**B**): Frequency graphs with the 15 most common words of each of the six clusters of Group B composed of the rest of the articles.

Figure 10 shows the most frequent words of the six clusters of Group A in the lefthand column and the most frequent words of the six clusters of Group B in the righthand column.

The six clusters of Group A have as their most frequent words “leaf” in Cluster 0; “soil” in Cluster 1; “flavonoid” and “pollen” in Cluster 2; “use”, “medicinal” and “plant” in Cluster 3; “fire”, “seed” and “germin” in Cluster 4; and “extract” and “active” in Cluster 5. The clusters of Group B have the most frequent main words “extract” in Cluster 0; “soil” in Cluster 1, “pollen” in Cluster 2; “oil” and “essenti” in Cluster 3; “abstract” and “avail” in Cluster 4; and “seed”, “speci” and “germin” in Cluster 5.

The greatest differences between the keywords of each cluster are found in the words “leaf” and “flavonoid” present in Clusters 0 and 2 of Group A, respectively. This deeper analysis reveals that in the set of most cited articles, there are clusters whose main themes differ from the clusters of the group of least cited articles. However, this variability is not great, and some differences can also be attributed to other factors, such as the different numbers of articles between the groups. According to this analysis, there are no significant thematic differences between the most and the least cited papers, suggesting that the common habit of selecting the most cited papers is not truly justified.

#### 2.1.5. Patents Related to the Genus Cistus

Patents are undoubtedly an invaluable source of information regarding the state-of-the art in an investigation. However, the vast majority of the reviews and scientific manuscripts completely neglect patents and do not include any reference to their state-of-the-art. In this study, information was compiled on all the patents in the Espacenet database that contain the term “*Cistus*” in their title or abstract. Espacenet is a project developed by the European Patent Office together with the Member States of the European Patent Organization that includes all the patents granted worldwide. The information obtained was processed using artificial intelligence applied to language processing in a process similar to that previously carried out with the articles compiled in the previous sections.

**Figure 11:**
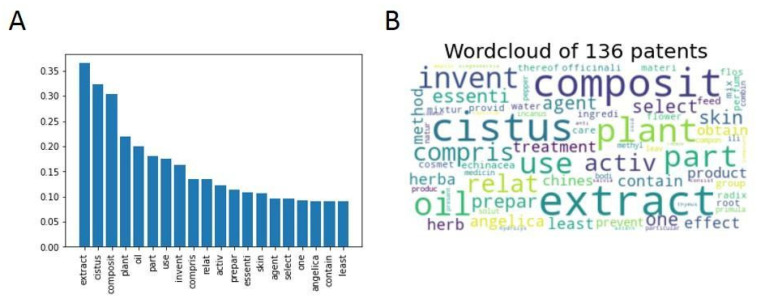
(**A**) Bar graph with the relative frequencies of the 20 most abundant words in the analyzed patents. (**B**) Word cloud as a visual representation of the frequency of the 75 most common words. A larger font size indicates a higher relative frequency of occurrence.

A total of 136 patents related to the genus *Cistus* were collected from Espacenet and analyzed. Figure 11 shows the most frequent words present in the collected patents.

Ignoring the high frequency of the terms “*Cistus*” and “plant”, a high prevalence of terms related to the production of extracts and oils, as well as their composition, can be observed. These elements are key to the patentability of products based on plants of the *Cistus* genus.

**Figure 12:**
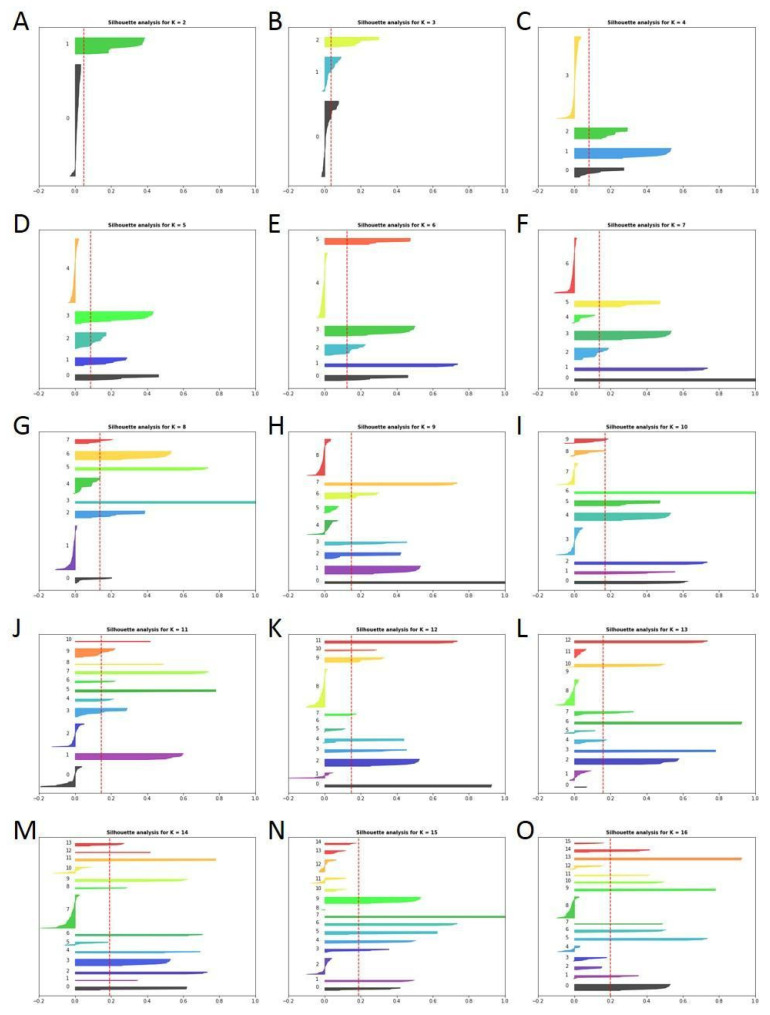
Silhouette plots for the different cluster numbers tested for the *Cistus* patent dataset. (**A**–**O**) show the results for grouping the articles in K groups (K value for each figure is shown in the upper part of each plot).

The silhouette score method was used to cluster the patents. Figure 12 shows the silhouette graphs for the separation of 2 into 16 different clusters.

**Figure 13:**
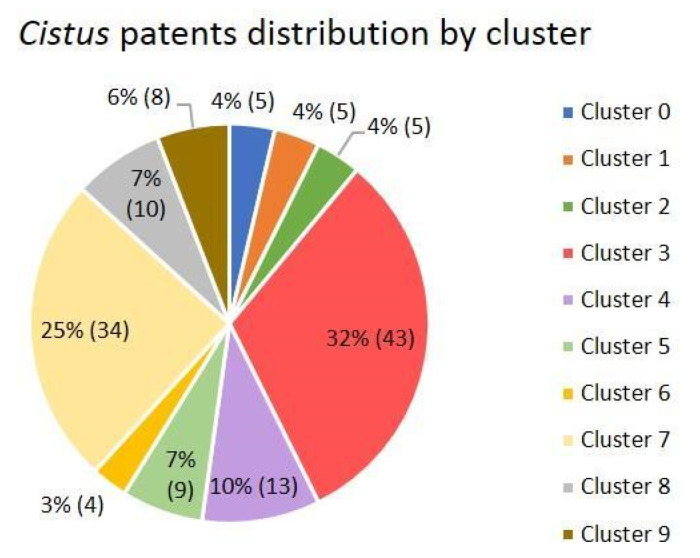
Pie chart with the percentage distribution of patents in each of the ten clusters presented as a percentage and as a total number in parentheses. Percentages in the pie chart have been rounded out until no decimals are shown in the figure to make the figure more simple.

It was estimated that ten was the optimal number of clusters to divide the dataset, and the clusters were numbered from 0 to 9. Figure 13 presents the number of patents included in all the clusters. The number of clusters was selected based on the silhouette graphs, as in the previous sections. Notably, the great difference between the silhouette lengths of scientific manuscript clustering (Figure 2) and the patents (Figure 12) suggests that patents are more case-specific than scientific manuscripts, which used to take a broader and less specific point of view.

**Figure 14:**
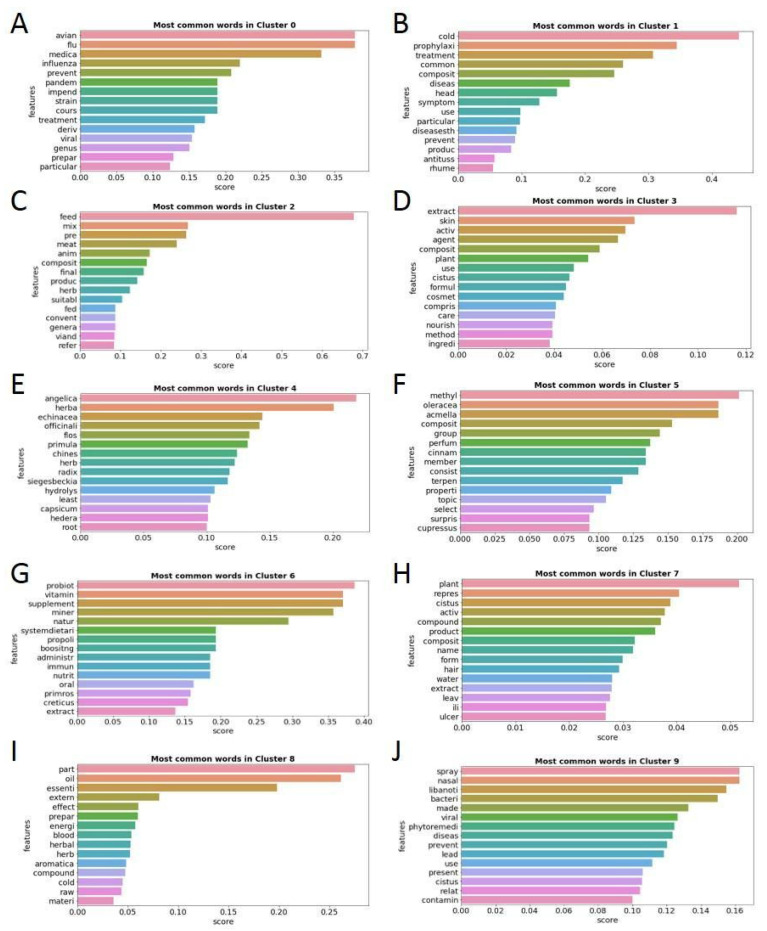
Fifteen words with the highest relative frequency of appearance in each of the ten patent clusters. Each cluster is identified on the top of each figure (**A–J**).

To gain an in-depth understanding of the theme of each cluster, a word frequency analysis was performed (Figure 14).

The predominant themes in each of the ten patent clusters obtained based on the most frequent words are the following: prevention and treatment of avian flu and influenza in subcluster 0, prophylaxis and treatment of colds and their symptoms in Cluster 1, mix of animal feed for better meat in subcluster 2, skin cosmetic uses of *Cistus* extracts in Cluster 3, different uses of plant extract hydrolysates in Cluster 4, perfume-related applications in Cluster 5, uses as natural health-boosting supplements in Cluster 6, diverse applications of *Cistus* and its parts, as well as specific records of plant varieties, in Cluster 7, essential oil applications in Cluster 8, and use of extracts in nasal spray form to prevent and treat viral and bacterial oropharyngeal infections in Cluster 9.

Cluster 3 groups the largest number of patents, with 32% of the total, and its main theme is the use of *Cistus* extracts as an active agent in cosmetic formulations for skin application. In general, the patents related to the *Cistus* genus are very diverse and include varied applications. The most common main topics in the patents are the use of extracts and essential oils for cosmetic applications and the prevention and treatment of viral infections. Other important registered uses are the use of extracts as health-boosting supplements, as cleaning agents, in perfumes or as part of animal feed.

#### 2.1.6. Article Subclustering

Owing to the clustering of all the articles available in Scopus related to plants of the *Cistus* genus obtained in Section 2.1.1, it is possible to select the most interesting set of articles for each field of research. In this case, Cluster 1, renamed the *Cistus* bioactivity cluster, was selected. This cluster includes articles related to the bioactive capacity of extracts and essential oils obtained from plants of the *Cistus* genus. The structure of the system’s programming enables selecting a specific cluster and reclustering its articles to achieve an even more specific thematic division. Cluster 1, made up of 489 different articles, was subjected to the same process of artificial intelligence applied to language processing to obtain and analyze the most relevant articles related to the antibacterial capacity of *Cistus*.

**Figure 15:**
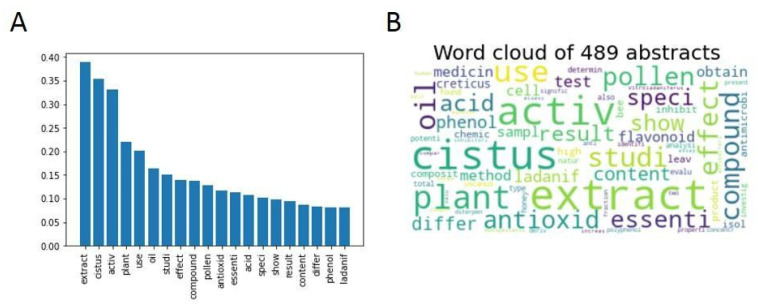
(**A**) Bar graph with the relative frequencies of the 20 most abundant words in the articles from the *Cistus* bioactivity cluster. (**B**) Word cloud as a visual representation of the frequency of the 75 most common words from the *Cistus* bioactivity cluster. A larger font size indicates a higher relative frequency of occurrence.

Figure 15 compares the words with the highest frequency in the *Cistus* bioactivity cluster in the forms of graphs and word clouds.

**Figure 16:**
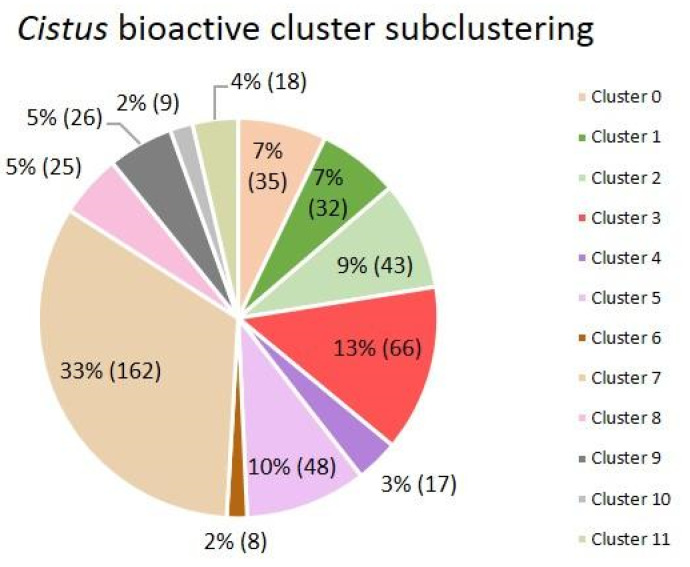
Pie chart with the distribution of articles in each of the subclusters presented as a percentage and as a total number in parentheses.

In this subclustering, it was determined that the optimal number of subclusters was 12, since it was observed that the distribution was the most homogeneous using the silhouette score method. The number of subclusters was selected based on the same criteria explained above (Supplementary Figure S5K). The distribution of articles by subcluster can be seen in Figure 16.

**Figure 17:**
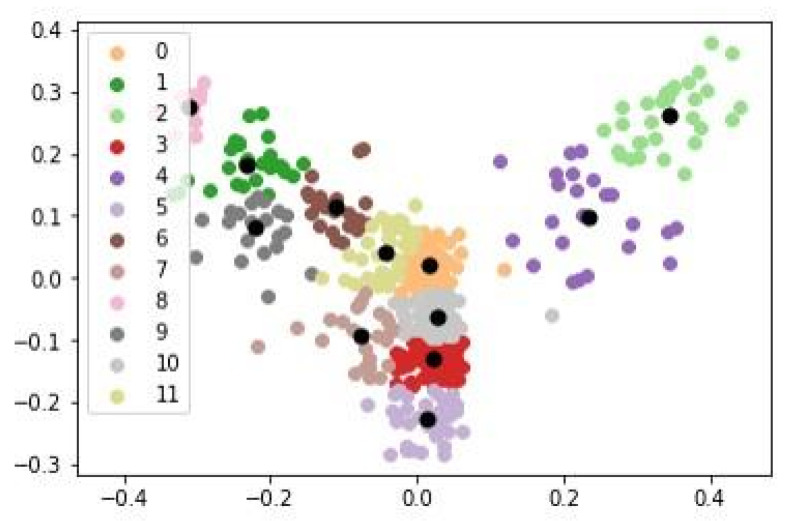
Scatter plot from PCA of the 489 articles present in the *Cistus* bioactivity cluster. The different colors represent the grouping of items in each of the 12 subclusters. The black dots represent the centroids of each cluster.

To visualize the complexity of the dataset and its clustering, PCA was used to obtain a 2D scatter plot showing the relationships among the different subclusters (Figure 17). In this figure, subclusters 0, 3, 5, 10, and 11 are closely related and are plotted in the central part.

**Figure 18:**
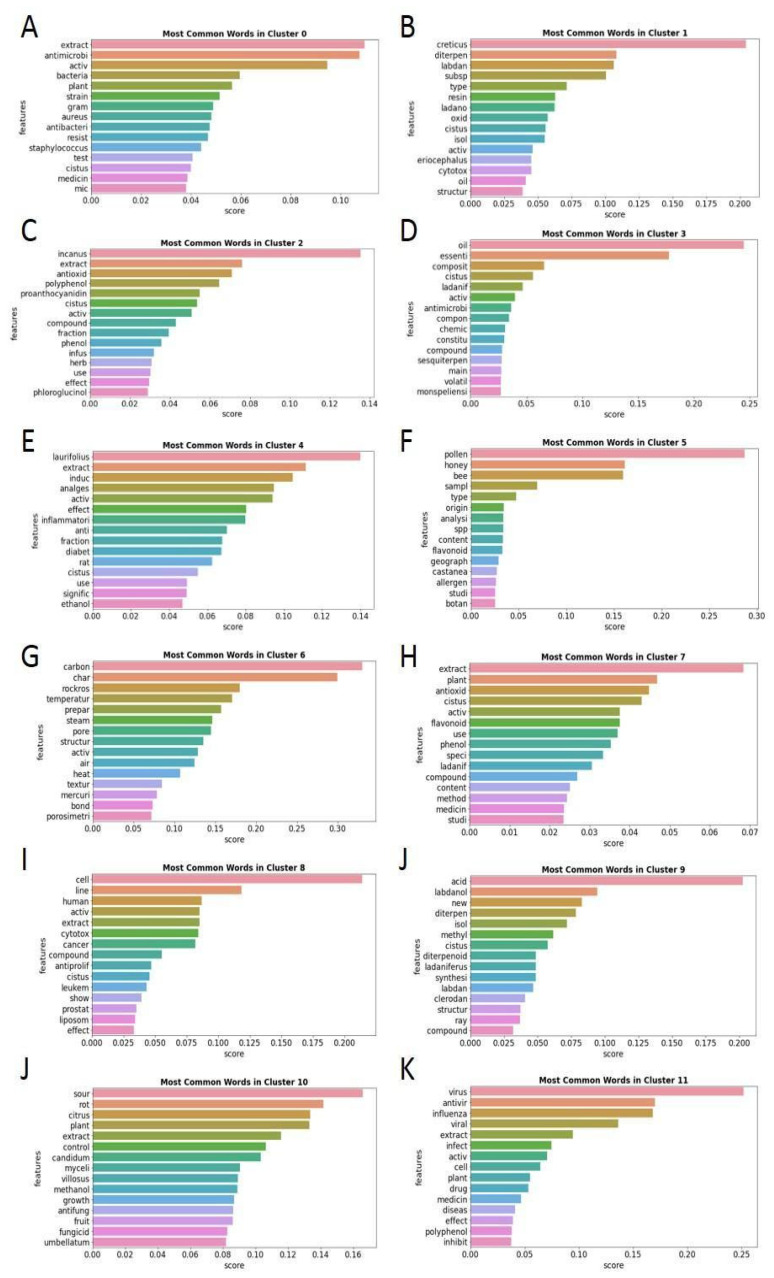
Fifteen words with the highest relative frequency of appearance in each of the 12 article subclusters. Each cluster is identified on the top of each figure (**A**–**K**).

The most common words of each of the 12 subclusters were analyzed to determine the research topic of the articles included in each of them. Figure 18 shows the 15 words with the highest relative frequency of appearance.

The most frequent words of each of the 12 subclusters give us an idea of the main theme of the articles present in each group. The main themes of each subcluster are antibacterial extracts in subcluster 0; *C. creticus* and its labdan diterpenes in subcluster 1; *C. incanus* extracts antioxidant bioactivity in subcluster 2; essential oils in subcluster 3; *C. laurifolius* extracts analgesic and anti-inflammatory bioactivities in subcluster 4; pollen, honey and bees in subcluster 5; activated carbon preparation using rockrose wood in subcluster 6; “antioxidant capacity of extracts” in subcluster 7; cell activity of extracts in subcluster 8; acids and labdan diterpenes in subcluster 9; antifungal activity against citrus sour rot in subcluster 10; and antiviral capacity of extracts in subcluster 11. As is clear from the topics included in each subcluster in Figure 17, the most related topics are close to each other. In this sense, antibacterial (subcluster 0), antiviral (subcluster 11) and antifungal (subcluster 10) activities are in the central part of the figure. Subcluster 7, related to antioxidant capacity, is also close to this core group, as most of the *Cistus* extracts contain bioactive compounds that present both antioxidant and antibacterial capacities, as previously stated [30]. On the other hand, the rest of the subclusters present a broader dispersion that can be interpreted as showing that they have lower coherence and minor relationships with the surrounding subclusters, which is completely coherent with the topics included in each of them that are described above.

Based on the results of these analyses, we can conclude that the most interesting articles for the aim of this work are found in subcluster 0, which groups the articles related to the antibacterial capacity of *Cistus* extracts. From the obtained table assigning each article to its corresponding subcluster, we can easily select the 35 articles that make up subcluster 0 and simply and objectively review the antibacterial capacity of the *Cistus* extracts.

#### 2.1.7. Article vs. Patent Comparison

As mentioned above, patents are usually forgotten in reviews of topics, leading to inaccurate summaries of researchers’ knowledge in those areas. Patents are usually considered nonscientific and more industry-related documents; however, they contain information about methodologies and results that normally complement the published scientific information. That is why reviewing patents is vital and should not be excluded from any scientific review.

In addition to the above developed analysis, we compared the 10 most cited words of the different groups obtained in some of the previous sections. When general scientific papers and patent groups (1601 papers and 136 patents) are compared, up to 5 words are common among the top 10: “activ”, “extract”, “use”, “*Cistus*” and “plant”. This comparison provides a general connection between both types of documents, which is clearly based on the biological activity of *Cistus* plants. This is the most interesting topic for industrial developments, as shown by the patents. The same five words are common when comparing the general groups of papers between Cluster 1 and derived subcluster 0. They are also common between Cluster 1 and subcluster 0 and among the general patent group and both Cluster 1 and subcluster 0. Finally, Cluster 1 and subcluster 0, both strongly related to the aim of this study (the antibacterial activity of *Cistus* plants), also share two additional words that confirm the accuracy of our analysis: “antimicrobi” and “medicin”.

However, despite their relevance for the scientific review process, patents have some drawbacks. Their main disadvantage is that they are often written in a very complex and redundant language that researchers perceive as difficult and somehow obscure. This is probably why researchers do not feel confident in reviewing patent documents. However, rather than a problem, this is an opportunity to increase our knowledge as scientists by learning and understanding how patents are written and how we can use the large amount of information that they already include. Another problem related to patents is that they are redundant and sometimes very similar to each other, making it necessary to take a deep look at them before accepting or discarding them as a document to be used in our reviews.

## 3. Antibacterial Capacity of Plants of the Genus *Cistus*

The abovementioned information indicates that the “real” review of the state-of-the-art on the use of extracts obtained from *Cistus* plants with antibacterial purposes begins here through the use of articles in subcluster 0 obtained from the scientific manuscripts present in Cluster 1 from article analysis 8 (Section 2.1.6) and Clusters 4 and 9 from the patent analysis (Section 2.1.5). Previous sections have provided a more objective selection of all available data, removing selection bias and providing insightful information about very interesting topics, such as the temporal trends within the topic, the open-access gap and citations.

There are many reports on the antibacterial capacity of *Cistus* extracts, which is measured using two main methods: microdilution (Table 1) and disk diffusion (Table 2).

The data presented in Table 1 suggest that the species with the most reports of activity are *C. munbyi* and *C. monspeliensis*, both with 27% of the total reports, followed by *C. creticus* with 23% of the total. For the parts most used to make the extracts, the leaves and the aerial parts are the most frequent, with 38% and 37% of the total, respectively. The most commonly used extraction solvent in these studies is ethanol, accounting for 22% of the total. It is followed by water at 17% and methanol at 16%.

In reference to the antibacterial capacity, the most commonly used bacteria in the tests are *S. aureus* with 27% of the total, *E. coli* with 17%, and *P. aeruginosa* with 11%. These data correspond to the susceptibility of these species to the different extracts. *S. aureus* was the species most sensitive to *Cistus* extracts by the microdilution method, with a mean MIC of 2.658 mg/mL. It is followed by *E. coli* with a mean MIC of 6.537 mg/mL and *P. aeruginosa* with a mean MIC of 7.765 mg/mL.

The data collected in Table 2 indicate that the most studied species in antibacterial disk diffusion assays are *C. creticus* with 37% of the total entries in the table and *C. monspeliensis* with 16%. The plant part most used to make the extracts is the leaves, representing 43% of the total, followed by the fruits with 23%. The most commonly used solvent is methanol, used in 19% of the studies, followed by butanol and water, both with 13%.

The most studied bacterial species in Table 2 is clearly *S. aureus,* with use in 39% of the total tests. It is followed by *B. cereus* with 14% and *B. subtilis* with 13%. As in Table 1, the percentage of use also coincides with its susceptibility to *Cistus* extracts. The most susceptible bacteria in the tests was *S. aureus* with a mean inhibition halo of 11.235 mm, followed by *B. cereus* with 10.270 mm and *B. subtilis* with 9.529 mm.

Comparing Table 1 and Table 2 results in some general conclusions: (1) *C. creticus* and *C. monspeliensis* are the most studied *Cistus* species for their antibacterial activity; (2) leaves are the most commonly used raw material; (3) alcoholic solvents are the most commonly used for extraction purposes; and (4) *S. aureus* is the most studied bacterial species, followed by some Gram-negative species such as *E. coli* and *P. aeruginosa* when the microdilution method is used (Table 1) and Gram-positive bacilli (*B. cereus* and *B. subtilis*) when the diffusion method is used (Table 2). On the other hand, it is clearly very difficult to select the best candidate, as the antibacterial activity is strongly influenced by the *Cistus* species, the bacteria tested, and the method employed; however, some *Cistus* species can be selected as the most active, including *C. salviifolius* and *C. cretitus*, as they have been tested against the longer list of bacteria.

The results obtained in this section are consistent with other studies that attribute a greater susceptibility of Gram-positive bacteria to different plant extracts [34,46]. These differences could be attributed to morphological and functional differences between Gram-positive and Gram-negative bacteria. It is possible that the presence of an external lipopolysaccharide layer and periplasmic space contribute to the greater resistance of gram-negative bacteria to plant extracts.

## 4. Methodology

Article information was downloaded from the Scopus website to one csv file. This file contains paragraphs on the abstract category and the articles’ meta-information, such as authors, journal, and year published, in the form of unstructured data. Since the goal of this analysis is to perform text analysis and clustering modeling, the focus was only on the columns [“Title”, “Year”, “Abstract”]. The “Abstract” and “Title” columns fetched from Scopus contained words in the format of sentences; therefore, the data required preprocessing prior to text analysis. First, the abstracts and title columns were extracted and connected. At this stage, unstructured text data were converted into normalized and structured data using the Python NLTK (Natural Language Toolkit) library.

Text data preprocessing involved three steps. The first step was noise removal, which included data cleaning of the text corpus. This was performed with the processCorpus function, which has the following steps: lowercase the text, remove all words that do not contribute to the semantic meaning of the text (words that are not within the English alphabet (e.g., e-mails and URLs), remove punctuation, remove numbers, remove generic stop words (common stop words in English include “the”, “a”, “is”, “what”, “will“, “you“, “and”, “but”, and “so”), remove custom stop words that we define (the list can include generic words that appear in the abstracts, such as “abstract“, “background“, “results“, and “conclusions“), remove any words composed of fewer than 2 or more than 21 letters and, finally, remove any excess white spaces.

The second step was text normalization, which refers to stemming. Stemming is a technique that reduces a word to its root. The root form is not necessarily a word by itself, but it can be used to generate words by concatenating the right suffix. For example, the words study, studies and studying stem into “studi”, which is not an English word. Nevertheless, it is permissible to keep all the remaining words in the simplest format possible, so a function that gives weights to each word can be applied.

The third step was tokenization, which was used to split sentences into words. The tokenized object is vectorized using the term frequency-inverse document frequency (TF-IDF) method. The TF-IDF is a statistical numerical feature used as a weighting factor that calculates the importance of words in each abstract relative to both what that abstract contains and all the other abstracts in the corpus. This normalization increases the importance of words that appear multiple times in the same abstract while decreasing the importance of words that appear in many abstracts (which would mostly be considered generic terms). TF-IDF gives each word in an abstract a score ranging from zero to one.

No prior knowledge regarding the groups of our abstracts was possessed. To identify structure in the unlabeled abstracts, unsupervised learning techniques were used. The K-means algorithm, a simple and popular unsupervised clustering algorithm employed to find groups of similar abstracts in a corpus, was used. The K-means algorithm calculates the distance between the points (abstracts in this case) and groups nearby abstracts together, indicating that they are similar. The K-means model was built with the K-means function in the Python sklearn library.

To evaluate the optimal number of clusters to choose when running the K-means algorithm using the given input parameters, the average silhouette method was used. It is estimated that a good cluster is one with a distance between the points within the cluster lower than the distance between points of two different clusters. Then, the top words in each cluster were examined, and the score of the best words in each cluster was observed. A map of words was created to allow researchers to visualize the text data clearly and simply by displaying the keywords that were closely related to each cluster concept.

Finally, the K-means algorithm generated cluster labels, which represented the abstracts contained in these clusters. To visualize the K-means clustering results, principal component analysis (PCA) was used to reduce the number of dimensions so that the results could be visualized using a 2D scatter plot. The trend of occurrence of each cluster was also plotted against the years in which the articles were published to obtain a time-based view of meaningful trends.

The Jupyter Notebook on Google Colab used in this work is available for free here. We hope that the dissemination of these tools will facilitate the work of other researchers in any field, promoting scientific progress and collaboration and enhancing global accessibility to scientific procedures in line with open science practices

## 5. Conclusions and Perspectives

Artificial intelligence applied to NLP has emerged as a powerful tool that can be applied to many aspects of our daily lives. In this manuscript, we have implemented and programmed a tool that allows to conduct an objective and efficient literature review using NLP. We have applied this methodology to all the existing bibliography of the genus *Cistus* to finally focus on the antibacterial activity of the *Cistus* plants. Although this approach has been already used in other areas of health sciences [47,48], we have built a complete Jupyter notebook on Google Colab to process, analyze, and select the most relevant bibliography, tool that can be implemented to any other area of scientific literature search. The output of the NLP-assisted literature review reveals that: (1) The most studied *Cistus* specie is *C. criticus,* followed by *C. monspeliensis* but up to nine different *Cistus* species present antibacterial results according to the bibliography. (2) Although studies using the whole plant and aerial parts are very common, those using fruits and especially leaves are the most frequent ones. (3) Different solvents are used for bioactive compounds extraction, but alcohols (ethanol, methanol, and butanol) and water are the most used. (4) *Cistus* plants extracts are especially active against Gram positive bacteria, mainly *S. aureus* but their activity is also significant against other Gram positive bacilli (*B. subtilis* and *B. cereus)* and Gram negative species as *E. coli* and *P. aeruginosa*. Furthermore, these bacterial species accumulate most of the studies using *Cistus* plant extracts.

Focusing on artificial intelligence applied to NLP, and comparing this methodology with traditional searches, it is not possible to efficiently review and check the relevance of 1601 scientific published papers and 136 patents, or, more important, do this in only a few minutes. Therefore, this new approach is truly significant, and the tools presented here should contribute to a paradigm change in how reviews and the state-of-the-art of scientific projects, papers and application documents are created. Artificial intelligence and NLP are here to stay, so we have to adapt to this new situation and use new methodologies to truly improve our outcomes.

However, artificial intelligence applied to NLP still needs human support to provide the results that we truly need for our research. In our case, human intervention is still needed to identify the key words to start with and select the number of clusters that truly represent the best distribution, as not only mathematical values should be taken into account. Human intervention is still needed in the final interpretation and cohesive analysis of the information. Our tool only provides the manuscript to be reviewed; the review process is still in human hands.

We also highlight the versatility of our tool, as it can be used for any topic and aim with a few simple modifications in the Python program. This is an extremely significant milestone because it will contribute to the open science policy that is specified by many regulatory and funding entities and promote the transparency of future research. In this sense, future studies, no matter their discipline, can use our tool to reduce their selection biases and increase transparency. Our work demonstrates that this tool is very versatile and suitable for application in any topic, providing not only the best selection of the text to be reviewed, but also giving temporal trends, information about citations and open access status. Furthermore, as it is above-mentioned, it can be used not only for scientific manuscript but also for project and grant applications.

Finally, the antibacterial activity of *Cistus* plants has been used as a proof of concept of the developed tool. In this sense, we have checked that the tool is truly and fully operative in a real situation, provides insightful results that confirm previous studies in the field, and helps us collect the main results in an easier and comprehensive way.

## Figures and Tables

**Table 1:** Antibacterial capacity of *Cistus* extracts measured by the microdilution method. The # symbol is used after the value when the value is MIC_50_ instead of MIC.

*Cistus* Species	Part Used	Extraction Solvent	Bacteria	Antibacterial Capacity MIC (mg/mL)	Reference
*C. salviifolius* L.	Flowers	Ethanol	*B. subtilis*	12.5	[31]
			*B. subtilis*	6.25	
			*E. coli*	12.5	
			*E. coli*	12.5	
			*L. monocytogenes*	6.25	
			*L. monocytogenes*	1.562	
	Leaves	Ethanol	*L. monocytogenes*	0.515	[31]
			*P. aeruginosa*	12.5	
			*P. aeruginosa*	12.5	
			*S. aureus*	3.125	
			*S. aureus*	1.562	
			*S. typhimurium*	12.5	
			*S. typhimurium*	12.5	[32]
		Methanol	*S. aureus*	4	[33]
			*S. aureus* ATCC 29213	8	
			*Staphylococcus epidermidis*	8	
		Water	Methicillin-resistant *S. aureus*	0.0512 #	[34,35]
			Methicillin-sensitive *S. aureus*	0.0807 #	
	Whole plant	Hydroalcoholic	*E. coli*	0.221 #	[36]
			*S. aureus*	0.050 #	
		Water	*E. coli*	0.245 #	
			*S. aureus*	0.0045 #	
*Cistus munbyi* Pomel	Aerial parts	Hexane	*Bacillus cereus* ATCC 11778	5	[37]
			*Bacillus subtilis* ATCC 6633	2.5	
			*Enterococcus faecalis* ATCC 29212	2.5	
			*Escherichia coli* ATCC 25922	2.5	
			*K. pneumoniae* ATCC 70603	2.5	
			*Klebsiella pneumoniae*	2.5	
			*Pseudomonas aeruginosa* ATCC 27835	2.5	
			*S. aureus*	5	
			*S. aureus* ATCC 25923	0.312	
			*S. aureus* ATCC 29213	5	
			*Salmonella montevideo* ATCC 3581	2.5	
		Chloroform	*B. cereus* ATCC 11778	2.5	[37]
			*B. subtilis* ATCC 6633	2.5	
			*E. coli* ATCC 25922	2.5	
			*E. faecalis* ATCC 29212	2.5	
			*K. pneumoniae*	0.625	
			*K. pneumoniae* ATCC 70603	5	
			*P. aeruginosa* ATCC 27835	5	
			*S. aureus*	2.5	
			*S. aureus* ATCC 25923	1.25	
			*S. aureus* ATCC 29213	0.625	
			*S. montevideo* ATCC 3581	2.5	
		Acetone	*B. cereus* ATCC 11778	1.25	[37]
			*B. subtilis* ATCC 6633	1.25	
			*E. coli* ATCC 25922	1.25	
			*E. faecalis* ATCC 29212	0.625	
			*K. pneumoniae* ATCC 70603	2.5	
			*P. aeruginosa* ATCC 27835	5	
			*S. aureus*	2.5	
			*S. aureus* ATCC 25923	2.5	
			*S. aureus* ATCC 29213	2.5	
			*S. montevideo* ATCC 3581	2.5	
		Ethanol	*B. cereus* ATCC 11778	2.5	[37]
			*B. subtilis* ATCC 6633	5	
			*E. coli* ATCC 25922	2.5	
			*E. faecalis* ATCC 29212	2.5	
			*K. pneumoniae* ATCC 70603	5	
			*P. aeruginosa* ATCC 27835	5	
			*S. aureus* ATCC 29213	2.5	
			*S. montevideo* ATCC 3581	2.5	
		Water	*E. faecalis* ATCC 29212	5	[37]
			*K. pneumoniae*	5	
			*P. mirabilis*	5	
			*P. mirabilis* ATCC 35659	5	
			*S. aureus*	5	
			*S. aureus* ATCC 25923	0.312	
			*S. aureus* ATCC 29213	2.5	
*Cistus ×incanus* L.	Aerial parts	Hydroalcoholic (45% Ethanol, 55% water)	*Borrelia burgdorferi*	0.25–0.5%	[38]
*Cistus creticus* L.	Aerial parts	Butanol	*E. coli* ATCC 8739	5	[39]
			*S. aureus* ATCC 6538	1.25	
		Ethyl acetate	*E. coli* ATCC 8739	1.25	[39]
			*S. aureus* ATCC 6538	2.5	
		Methanol	*Enterococcus faecalis* ATCC 29212	5	[40]
			*Fusobacterium nucleatum* ATCC 25586	0.6	
			*Parvimonas micra* ATCC 23195	0.08	
			*Porphyromonas gingivalis* W381	0.08	
			*Prevotella intermedia* MSP 34	0.3	
			*S. aureus* ATCC 25923	0.6	
			*Streptococcus mutans* DSM 20523	5	
			*Streptococcus oralis* ATCC 35037	0.3	
			*Streptococcus sobrinus* DSM 20381	5	
		Water	*E. coli* ATCC 8739	24.8	[39]
			*S. aureus* ATCC 6538	6.2	
	Flowers	Ethanol	*B. subtilis*	0.25	[41]
			*E. coli*	0.5	
			*S. aureus*	0.25	
		Acetone	*B. subtilis*	0.5	[41]
			*E. coli*	1	
			*S. aureus*	0.25	
	Leaves	Ethanol	*B. subtilis*	0.125	[41]
			*S. aureus*	0.125	
		Butanol	*E. coli*	1.56	[42]
			*E. hirea*	1.56	
			*P. aeruginosa*	3.125	
			*S. aureus*	0.78	
		Ethyl acetate	*E. coli*	1.56	[42]
			*Enterococcus hirea*	1.56	
			*P. aeruginosa*	3.125	
			*S. aureus*	1.56	
		Methanol	*E. coli*	3.125	[42]
			*E. hirea*	3.125	
			*P. aeruginosa*	3.125	
			*S. aureus*	0.78	
		Water	*E. coli*	3.125	[42]
			*E. hirea*	6.25	
			*P. aeruginosa*	3.125	
			*S. aureus*	1.56	
*Cistus monspeliensis* L.	Flowers	Ethanol	*B. subtilis*	12.5	[31]
			*E. coli*	12.5	
			*L. monocytogenes*	1.562	
			*P. aeruginosa*	12.5	
			*S. aureus*	1.562	
			*S. typhimurium*	12.5	
	Leaves	Acetone	*E. faecalis*	1.25	[43]
			*P. aeruginosa*	1.25	
			*S. aureus*	0.156	
			*S. epidermidis*	0.312	
			*S. saprophiticus*	0.312	
		Butanol	*E. coli*	3.125	[42]
			*E. hirea*	1.56	
			*P. aeruginosa*	1.56	
			*S. aureus*	1.56	
		Ethanol	*B. subtilis*	12.5	[31]
			*E. coli*	12.5	
			*L. monocytogenes*	3.125	
			*P. aeruginosa*	12.5	
			*S. aureus*	1.562	
			*S. typhimurium*	12.5	
		Ethyl acetate	*E. coli*	3.125	[42]
			*Enterococcus hirea*	3.125	
			*P. aeruginosa*	6.25	
			*S. aureus*	1.56	
		Hexane	*S. aureus*	0.625	[43]
			*S. epidermidis*	1.25	
			*S. saprophiticus*	1.25	
		Methanol	*E. coli*	25	[43]
			*E. faecalis*	1.25	[42]
			*E. hirea*	25	[43]
			*P. aeruginosa*	0.625	[42]
			*P. aeruginosa*	50	
			*S. aureus*	0.312	[43]
			*S. aureus*	25	[42]
			*S. epidermidis*	0.312	[43]
			*S. saprophiticus*	0.625	
		Water	*E. coli*	6.25	[42]
			*E. hirea*	6.25	
			*P. aeruginosa*	6.25	
			*S. aureus*	3.125	
	Whole plant	Hydroalcoholic (80% Methanol, 20% water)	*E. cloacae*	1.6	[44]
			*E. coli*	1.6	
			*E. faecalis*	1.6	
			*K. pneumoniae*	3.2	
			*P. aeruginosa*	1.6	
			*S. aureus*	0.2	
*Cistus ladanifer* L.	Whole plant	Hydroalcoholic	*E. coli*	0.113 #	[36]
			*S. aureus*	0.144 #	
		Water	*E. coli*	0.248 #	[36]
			*S. aureus*	0.151 #	
*Cistus albidus* L.	Whole plant	Hydroalcoholic	*E. coli*	0.233 #	[36]
			*S. aureus*	0.292 #	
		Water	*E. coli*	0.336 #	[36]
			*S. aureus*	0.060 #	
*Cistus clusii* Dunal	Whole plant	Hydroalcoholic	*E. coli*	0.116 #	[36]
			*S. aureus*	0.304 #	
		Water	*E. coli*	0.501 #	[36]
			*S. aureus*	0.091 #	
*Cistus laurifolius* L.	Flowers	Butanol	*Helicobacter pylori*	0.00195	[45]
		Chloroform	*H. pylori*	0.125	[45]
		Methanol	*H. pylori*	0.625	[45]
		Water	*H. pylori*	0.625	[45]

# is used after the value when the value is MIC_50_ instead of MIC.

**Table 2:** Antibacterial capacity of *Cistus* extracts measured by the disk diffusion method.

*Cistus* Species	Part Used	Extraction Solvent	Bacteria	Antibacterial Capacity MIC (mg/mL)	Reference
*C. salviifolius* L.	Flowers	Ethanol	*B. subtilis*	12.5	[31]
			*B. subtilis*	6.25	
			*E. coli*	12.5	
			*E. coli*	12.5	
			*L. monocytogenes*	6.25	
			*L. monocytogenes*	1.562	
	Leaves	Ethanol	*L. monocytogenes*	0.515	[31]
			*P. aeruginosa*	12.5	
			*P. aeruginosa*	12.5	
			*S. aureus*	3.125	
			*S. aureus*	1.562	
			*S. typhimurium*	12.5	
			*S. typhimurium*	12.5	[32]
		Methanol	*S. aureus*	4	[33]
			*S. aureus* ATCC 29213	8	
			*Staphylococcus epidermidis*	8	
		Water	Methicillin-resistant *S. aureus*	0.0512 #	[34,35]
			Methicillin-sensitive *S. aureus*	0.0807 #	
	Whole plant	Hydroalcoholic	*E. coli*	0.221 #	[36]
			*S. aureus*	0.050 #	
		Water	*E. coli*	0.245 #	[36]
			*S. aureus*	0.0045 #	
*Cistus munbyi* Pomel	Aerial parts	Hexane	*Bacillus cereus* ATCC 11778	5	[37]
			*Bacillus subtilis* ATCC 6633	2.5	
			*Enterococcus faecalis* ATCC 29212	2.5	
			*Escherichia coli* ATCC 25922	2.5	
			*K. pneumoniae* ATCC 70603	2.5	
			*Klebsiella pneumoniae*	2.5	
			*Pseudomonas aeruginosa* ATCC 27835	2.5	
			*S. aureus*	5	
			*S. aureus* ATCC 25923	0.312	
			*S. aureus* ATCC 29213	5	
			*Salmonella montevideo* ATCC 3581	2.5	
		Chloroform	*B. cereus* ATCC 11778	2.5	[37]
			*B. subtilis* ATCC 6633	2.5	
			*E. coli* ATCC 25922	2.5	
			*E. faecalis* ATCC 29212	2.5	
			*K. pneumoniae*	0.625	
			*K. pneumoniae* ATCC 70603	5	
			*P. aeruginosa* ATCC 27835	5	
			*S. aureus*	2.5	
			*S. aureus* ATCC 25923	1.25	
			*S. aureus* ATCC 29213	0.625	
			*S. montevideo* ATCC 3581	2.5	
		Acetone	*B. cereus* ATCC 11778	1.25	[37]
			*B. subtilis* ATCC 6633	1.25	
			*E. coli* ATCC 25922	1.25	
			*E. faecalis* ATCC 29212	0.625	
			*K. pneumoniae* ATCC 70603	2.5	
			*P. aeruginosa* ATCC 27835	5	
			*S. aureus*	2.5	
			*S. aureus* ATCC 25923	2.5	
			*S. aureus* ATCC 29213	2.5	
			*S. montevideo* ATCC 3581	2.5	
		Ethanol	*B. cereus* ATCC 11778	2.5	[37]
			*B. subtilis* ATCC 6633	5	
			*E. coli* ATCC 25922	2.5	
			*E. faecalis* ATCC 29212	2.5	
			*K. pneumoniae* ATCC 70603	5	
			*P. aeruginosa* ATCC 27835	5	
			*S. aureus* ATCC 29213	2.5	
			*S. montevideo* ATCC 3581	2.5	
		Water	*E. faecalis* ATCC 29212	5	[37]
			*K. pneumoniae*	5	
			*P. mirabilis*	5	
			*P. mirabilis* ATCC 35659	5	
			*S. aureus*	5	
			*S. aureus* ATCC 25923	0.312	
			*S. aureus* ATCC 29213	2.5	
*Cistus ×incanus* L.	Aerial parts	Hydroalcoholic (45% ethanol, 55% water)	*Borrelia burgdorferi*	0.25–0.5%	[38]
*Cistus creticus* L.	Aerial parts	Butanol	*E. coli* ATCC 8739	5	[39]
			*S. aureus* ATCC 6538	1.25	
		Ethyl acetate	*E. coli* ATCC 8739	1.25	[39]
			*S. aureus* ATCC 6538	2.5	
		Methanol	*Enterococcus faecalis* ATCC 29212	5	[40]
			*Fusobacterium nucleatum* ATCC 25586	0.6	
			*Parvimonas micra* ATCC 23195	0.08	
			*Porphyromonas gingivalis* W381	0.08	
			*Prevotella intermedia* MSP 34	0.3	
			*S. aureus* ATCC 25923	0.6	
			*Streptococcus mutans* DSM 20523	5	
			*Streptococcus oralis* ATCC 35037	0.3	
			*Streptococcus sobrinus* DSM 20381	5	
		Water	*E. coli* ATCC 8739	24.8	[39]
			*S. aureus* ATCC 6538	6.2	
	Flowers	Ethanol	*B. subtilis*	0.25	[41]
			*E. coli*	0.5	
			*S. aureus*	0.25	
		Acetone	*B. subtilis*	0.5	[41]
			*E. coli*	1	
			*S. aureus*	0.25	
	Leaves	Ethanol	*B. subtilis*	0.125	[41]
			*S. aureus*	0.125	
		Butanol	*E. coli*	1.56	[42]
			*E. hirea*	1.56	
			*P. aeruginosa*	3.125	
			*S. aureus*	0.78	
		Ethyl acetate	*E. coli*	1.56	[42]
			*Enterococcus hirea*	1.56	
			*P. aeruginosa*	3.125	
			*S. aureus*	1.56	
		Methanol	*E. coli*	3.125	[42]
			*E. hirea*	3.125	
			*P. aeruginosa*	3.125	
			*S. aureus*	0.78	
		Water	*E. coli*	3.125	[42]
			*E. hirea*	6.25	
			*P. aeruginosa*	3.125	
			*S. aureus*	1.56	
*Cistus monspeliensis* L.	Flowers	Ethanol	*B. subtilis*	12.5	[31]
			*E. coli*	12.5	
			*L. monocytogenes*	1.562	
			*P. aeruginosa*	12.5	
			*S. aureus*	1.562	
			*S. typhimurium*	12.5	
	Leaves	Acetone	*E. faecalis*	1.25	[43]
			*P. aeruginosa*	1.25	
			*S. aureus*	0.156	
			*S. epidermidis*	0.312	
			*S. saprophiticus*	0.312	
		Butanol	*E. coli*	3.125	[42]
			*E. hirea*	1.56	
			*P. aeruginosa*	1.56	
			*S. aureus*	1.56	
		Ethanol	*B. subtilis*	12.5	[31]
			*E. coli*	12.5	
			*L. monocytogenes*	3.125	
			*P. aeruginosa*	12.5	
			*S. aureus*	1.562	
			*S. typhimurium*	12.5	
		Ethyl acetate	*E. coli*	3.125	[42]
			*Enterococcus hirea*	3.125	
			*P. aeruginosa*	6.25	
			*S. aureus*	1.56	
		Hexane	*S. aureus*	0.625	[43]
			*S. epidermidis*	1.25	
			*S. saprophiticus*	1.25	
		Methanol	*E. coli*	25	[43]
			*E. faecalis*	1.25	[42]
			*E. hirea*	25	[43]
			*P. aeruginosa*	0.625	[42]
			*P. aeruginosa*	50	
			*S. aureus*	0.312	[43]
			*S. aureus*	25	[42]
			*S. epidermidis*	0.312	[43]
			*S. saprophiticus*	0.625	
		Water	*E. coli*	6.25	[42]
			*E. hirea*	6.25	
			*P. aeruginosa*	6.25	
			*S. aureus*	3.125	
	Whole plant	Hydroalcoholic (80% methanol, 20% water)	*E. cloacae*	1.6	[44]
			*E. coli*	1.6	
			*E. faecalis*	1.6	
			*K. pneumoniae*	3.2	
			*P. aeruginosa*	1.6	
			*S. aureus*	0.2	
*Cistus ladanifer* L.	Whole plant	Hydroalcoholic	*E. coli*	0.113 #	[36]
			*S. aureus*	0.144 #	
		Water	*E. coli*	0.248 #	[36]
			*S. aureus*	0.151 #	
*Cistus albidus* L.	Whole plant	Hydroalcoholic	*E. coli*	0.233 #	[36]
			*S. aureus*	0.292 #	
		Water	*E. coli*	0.336 #	[36]
			*S. aureus*	0.060 #	
*Cistus clusii* Dunal	Whole plant	Hydroalcoholic	*E. coli*	0.116 #	[36]
			*S. aureus*	0.304 #	
		Water	*E. coli*	0.501 #	[36]
			*S. aureus*	0.091 #	
*Cistus laurifolius* L.	Flowers	Butanol	*Helicobacter pylori*	0.00195	[45]
		Chloroform	*H. pylori*	0.125	[45]
		Methanol	*H. pylori*	0.625	[45]
		Water	*H. pylori*	0.625	[45]

# is used after the value when the value is MIC_50_ instead of MIC

## Data Availability

The Jupyter notebook used in this work can be found for free at https://drive.google.com/file/d/10qWGY3mCQQ-xg5aP6EGDNqy8ba1Er_01/view?usp=sharing, accessed on 21 May 2022.

## Supplementary Materials

The following supporting information can be downloaded at: https://www.mdpi.com/article/10.3390/antibiotics12020327/s1, Figure S1: Silhouette plots for the different cluster numbers tested for the Open Access Cistus articles dataset up to 16; Figure S2: Silhouette plots for the different cluster numbers tested for the non-Open Access Cistus articles dataset up to 16; Figure S3: Silhouette plots for the different cluster numbers tested for the top 20% most cited Cistus articles dataset up to 16; Figure S4: Silhouette plots for the different cluster numbers tested for the 80% least cited Cistus articles dataset up to 16; Figure S5: Silhouette plots for the different subcluster numbers tested for the bioactive Cluster 1 dataset up to 16.
