# Supplementary material for: Artificial Intelligence Applied to Improve Scientific Reviews: The Antibacterial Activity of Cistus Plants as Proof of Concept
Source: Antibiotics (Basel). 2023 Feb 4;12(2):327. doi: 10.3390/antibiotics12020327 (PMC9952093; doi:10.3390/antibiotics12020327)
Supplement: Supplementary file 1 [file antibiotics-12-00327-s001.zip › antibiotics-2173597-supplementary.pdf]

antibiotics

Article

Artificial Intelligence Applied to Improve Scientific Reviews: The Antibacterial Activity of *Cistus* Plants as Proof of Concept

Francisco Javier Álvarez-Martínez [1], Fernando Borrás-Rocher [2], Vicente Micol [1,3,*,†] and Enrique Barrajón-Catalán [1,4,†]

1 Instituto de Investigación, Desarrollo e Innovación en Biotecnología Sanitaria de Elche (IDiBE), Universidad Miguel Hernández (UMH), 03202 Elche, Spain
2 Statistics and Operative Research Department, UMH, Avda, Universidad s/n, 03202 Elche, Spain
3 CIBER, Fisiopatología de la Obesidad y la Nutrición, CIBERobn, Instituto de Salud Carlos III (CB12/03/30038), 28029 Madrid, Spain
4 Department of Pharmacy, Elche University Hospital-FISABIO, 03203 Elche, Spain
* Correspondence: vmicol@umh.es; Tel.: +34-965-222-586
† Both authors share senior authorship.

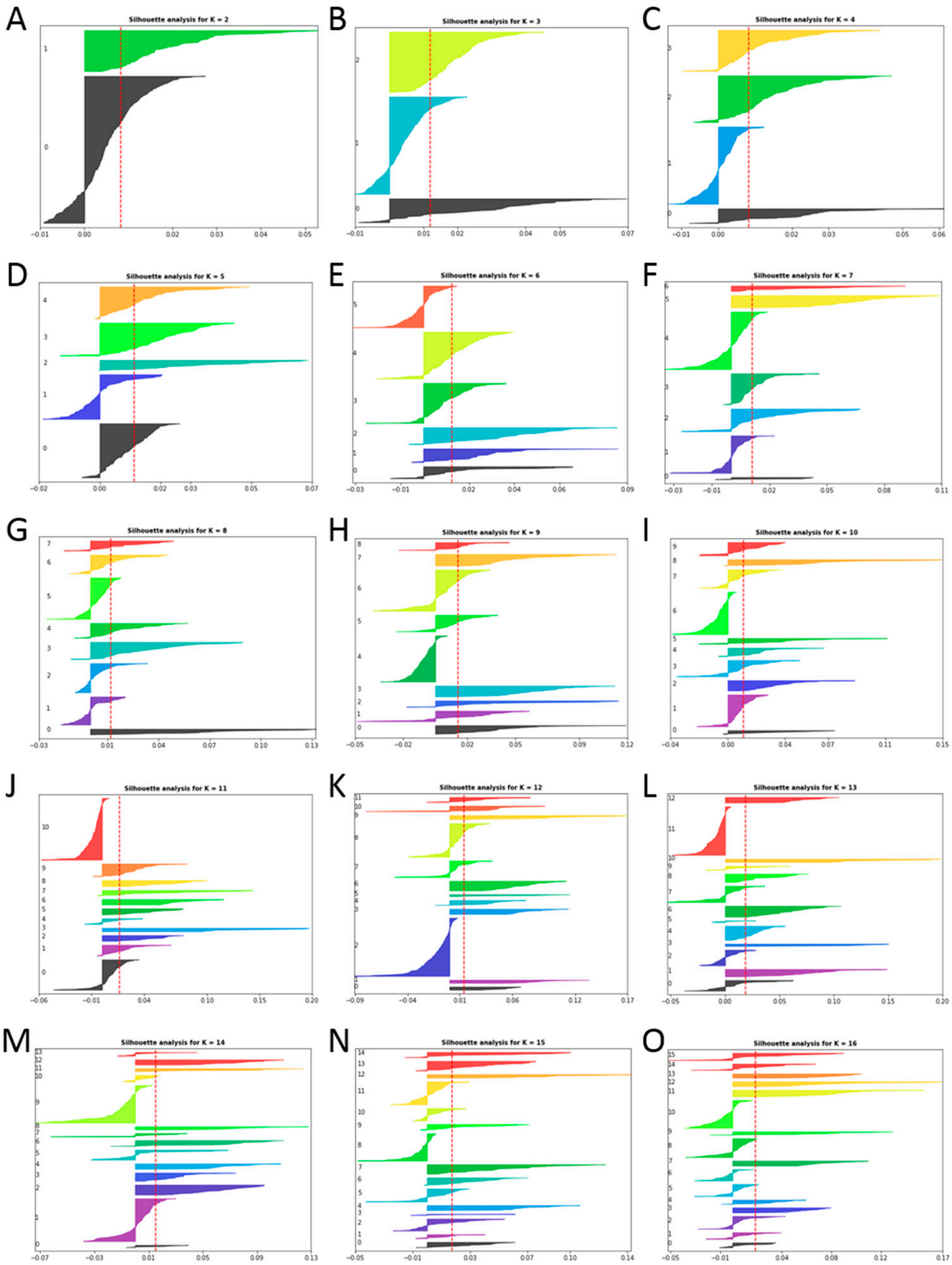

Figure S1. Silhouette plots for the different cluster numbers tested for the Open Access *Cistus* articles dataset up to 16 (**A–O**).

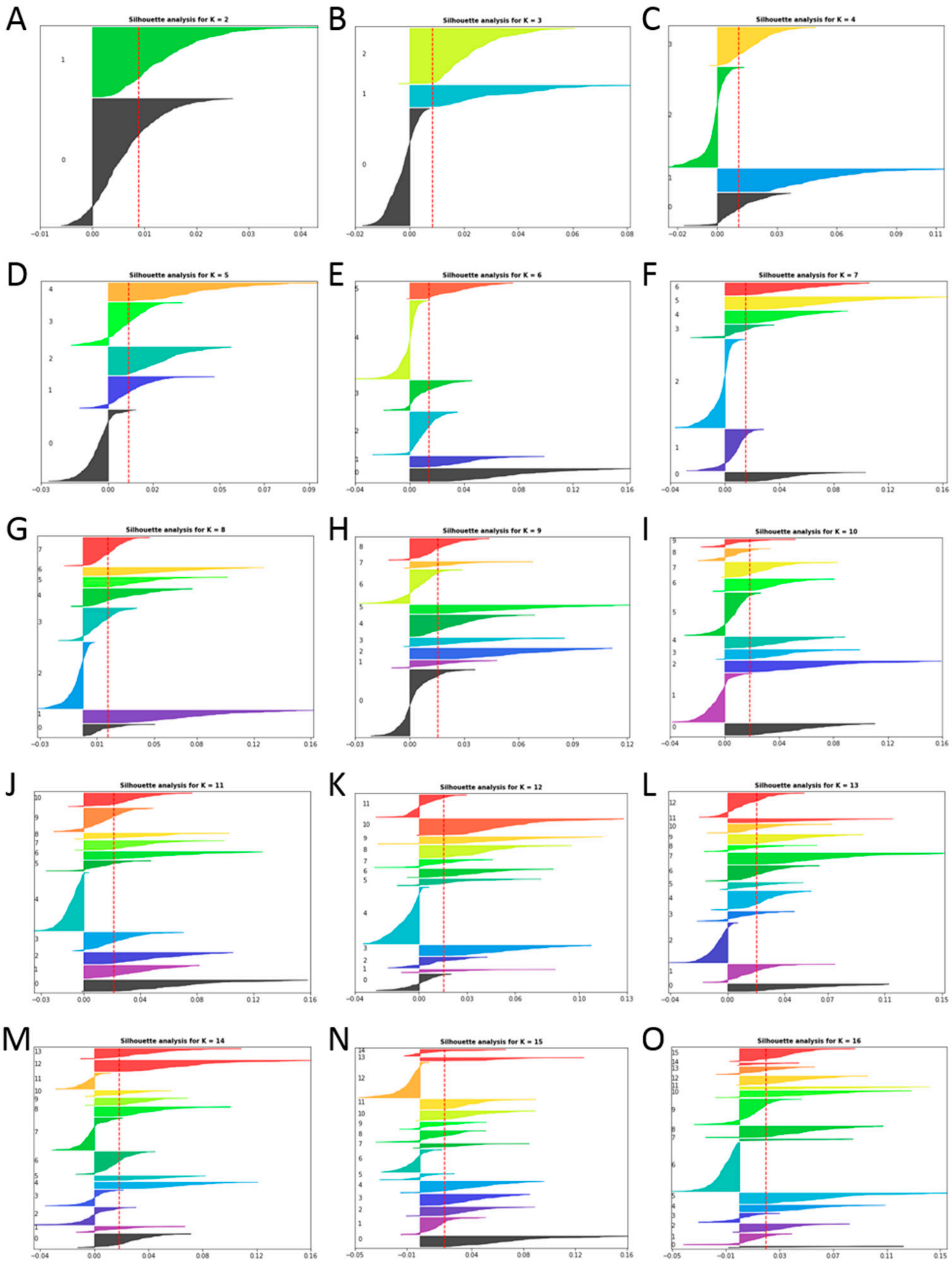

Figure S2. Silhouette plots for the different cluster numbers tested for the non-Open Access *Cistus* articles dataset up to 16 (**A–O**).

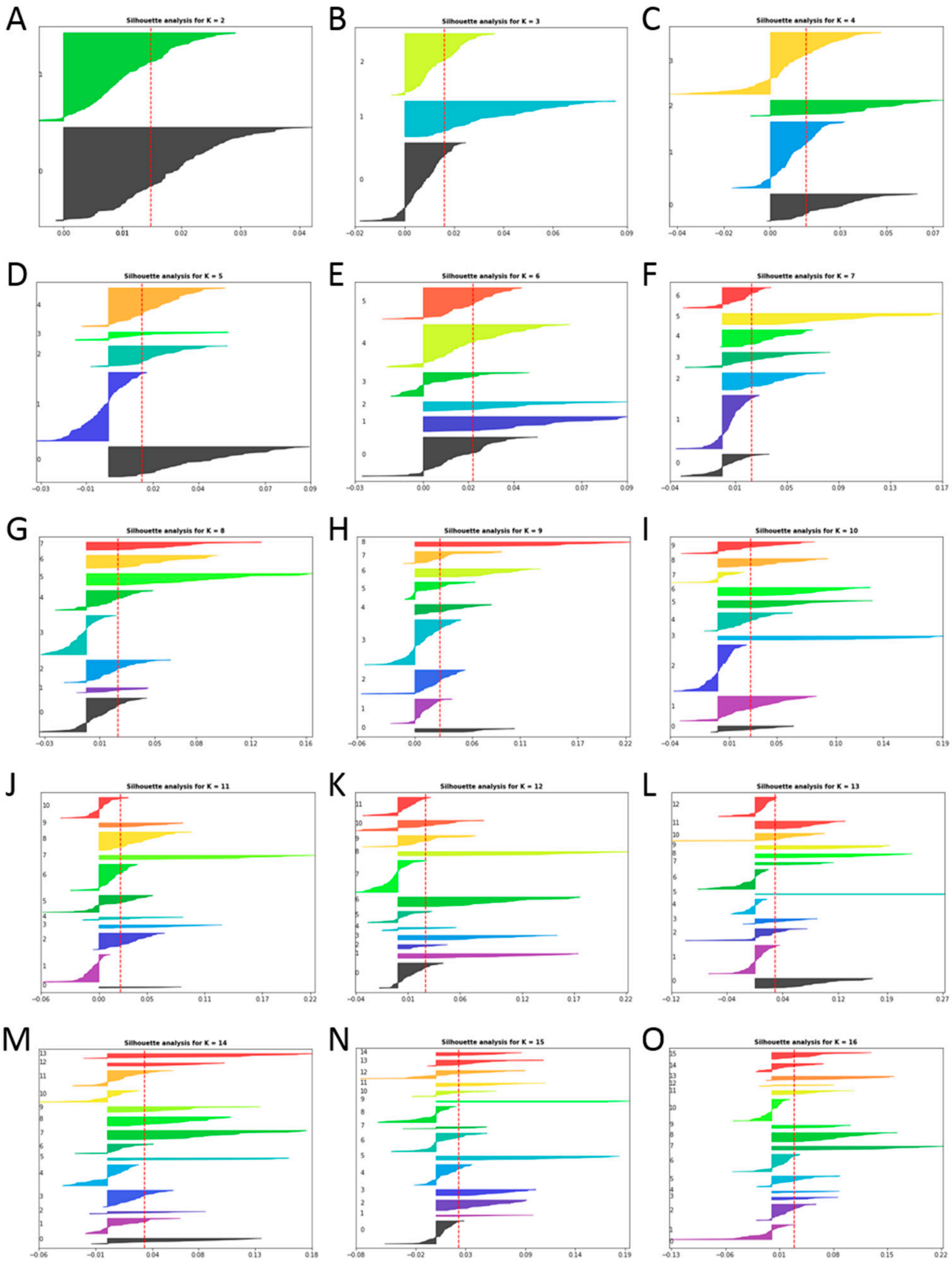

Figure S3. Silhouette plots for the different cluster numbers tested for the top 20% most cited *Cistus* articles dataset up to 16 (**A–O**).

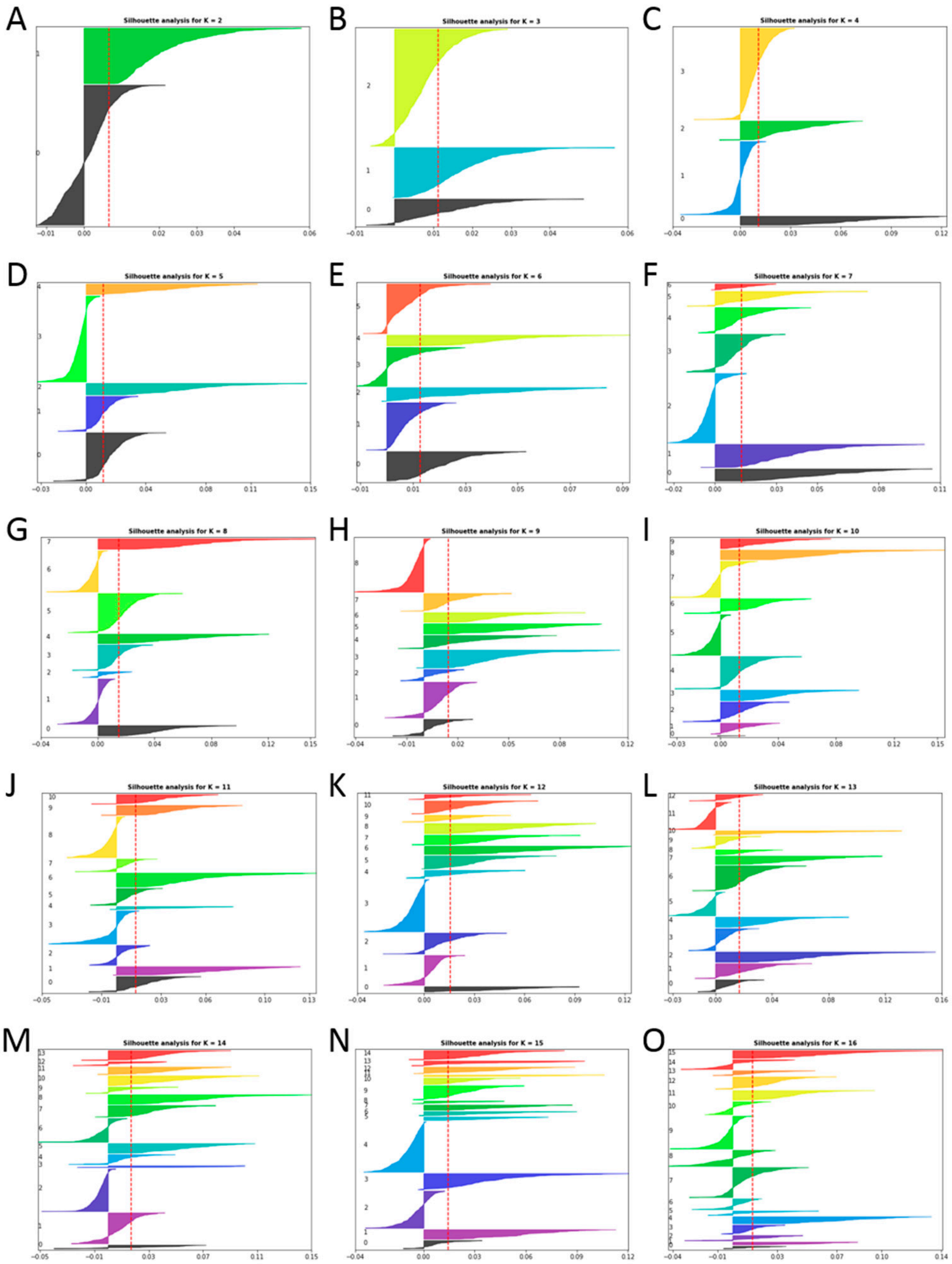

Figure S4. Silhouette plots for the different cluster numbers tested for the 80% least cited *Cistus* articles dataset up to 16 (**A–O**).

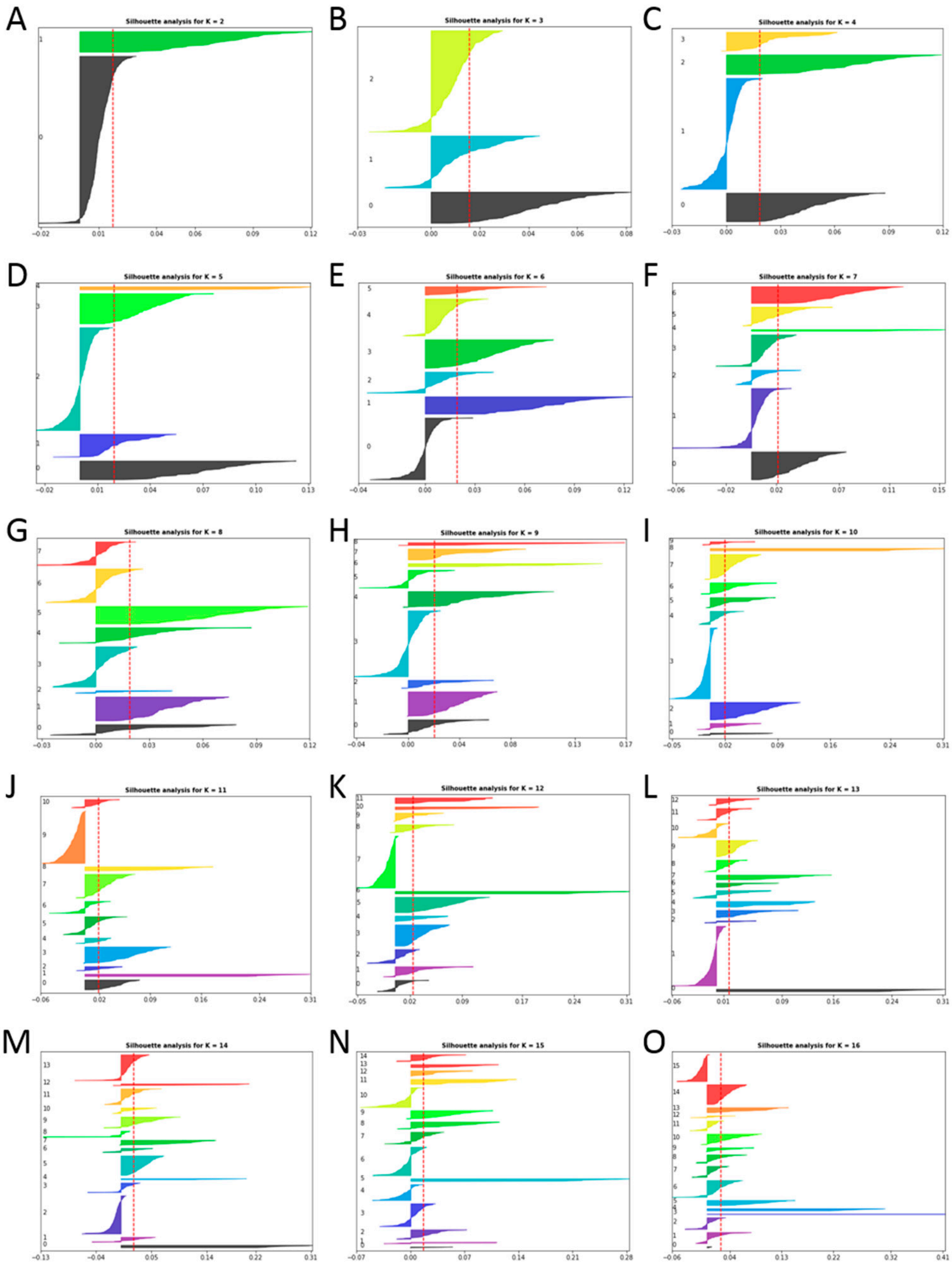

Figure S5. Silhouette plots for the different subcluster numbers tested for the bioactive Cluster 1 dataset up to 16 (**A–O**).
